# Amn1 governs post-mitotic cell separation in *Saccharomyces cerevisiae*

**DOI:** 10.1371/journal.pgen.1007691

**Published:** 2018-10-01

**Authors:** Ou Fang, Xiaohua Hu, Lin Wang, Ning Jiang, Jixuan Yang, Bo Li, Zewei Luo

**Affiliations:** 1 Laboratory of Population and Quantitative Genetics, Institute of Biostatistics and Genetics, School of Life Sciences, Fudan University, Shanghai, China; 2 School of Biosciences, the University of Birmingham, Birmingham, United Kingdom; 3 Department of Evolution and Ecology, School of Life Sciences, Fudan University, Shanghai, China; Stanford University School of Medicine, UNITED STATES

## Abstract

Post-mitotic cell separation is one of the most prominent events in the life cycle of eukaryotic cells, but the molecular underpinning of this fundamental biological process is far from being concluded and fully characterized. We use budding yeast *Saccharomyces cerevisiae* as a model and demonstrate *AMN1* as a major gene underlying post-mitotic cell separation in a natural yeast strain, YL1C. Specifically, we define a novel 11-residue domain by which Amn1 binds to Ace2. Moreover, we demonstrate that Amn1 induces proteolysis of Ace2 through the ubiquitin proteasome system and in turn, down-regulates Ace2’s downstream target genes involved in hydrolysis of the primary septum, thus leading to inhibition of cell separation and clumping of haploid yeast cells. Using ChIP assays and site-specific mutation experiments, we show that Ste12 and the a1-α12 heterodimer are two direct regulators of *AMN1*. Specifically, a1-α2, a diploid-specific heterodimer, prevents Ste12 from inactivating *AMN1* through binding to its promoter. This demonstrates how the Amn1-governed cell separation is highly cell type dependent. Finally, we show that *AMN1*^*368D*^ from YL1C is a dominant allele in most strains of *S*. *cerevisiae* and evolutionarily conserved in both genic structure and phenotypic effect in two closely related yeast species, *K*. *lactis* and *C*. *glabrata*.

## Author summary

Separation of mother and daughter cells after mitosis in eukaryotes enacts various functional and/or developmental needs and has significant medical and industrial implications. How this cellular behaviour is regulated is far from being concluded. We report here a novel Amn1 mediated post-mitotic cell separation in a budding yeast strain, YL1C and demonstrate that the post-mitotic cell separation can be regulated through a ubiquitin-conjugated protein degradation of Ace2 by Amn1. The Amn1-governed switch of cell separation is evolutionarily conserved and highly cell type dependent. These findings provide insights into the molecular mechanism of how post-mitotic cell separation is regulated in budding yeast, and data for translating into medical and industrial applications.

## Introduction

The switch between effective and inhibited separation of mother and daughter cells in eukaryotic mitosis represents a fundamental process for understanding the evolution of organizational and functional complexity of organisms, and also has significant medical and industrial value [1–3]. It has been well documented that division of the cytoplasm in *Saccharomyces cerevisiae* is comprised of a series of coordinated events including assembly and contraction of the contractile actomyosin ring in mitosis, formation of the primary and secondary septa and finally separation of mother and daughter cells [1]. The molecular machinery and regulatory networks that underlie this process has been significantly advanced in recent studies in the simple eukaryotic model yeast *S*. *cerevisae*. In particular, the RAM (regulation of Ace2 and morphogenesis) network, a pathway regulated by the MEN (Mitotic Exit Network), has been proposed to be responsible for nuclear importation of the transcription factor Ace2, which is needed for septum cleavage and post-mitotic cell separation [4–7]. Nuclear importation of Ace2 drives a sharp increase in the transcription of genes involved in septum cleavage, including *CTS1* and *SCW11*, and ultimately leads to separation of mother and daughter cells [4, 5]. So far, six proteins have been identified as key nodes in the RAM network: Sog2, Tao3, Hym1, Kic1, Mob2 and Cbk1. Cells lacking Ace2 or any of these six core components show a phenotypic defect of indistinguishable post-mitotic cell separation and cell clumping [6, 7]. Failure of daughter cells to separate from their mothers after mitosis, showing a snowflake phenotype under the micropscope, is recognized as the early evolution from unicellular to multicellular populations and the transition can evolve quickly over the course of multiple rounds of selection for the snowflake phenotype [8–10].

In our earlier work, we dissected phenotypic variation in cell clumping in a segregating population created by crossing two phenotypically divergent strains (YL1C with a strong clumpy phenotype and YH1A with effective cell separation), into four major cell clumping Quantitative Trait Loci (QTLs) [11]. These major QTLs together explained 45% of the trait phenotypic variation. We resolved the major QTL explaining 25% of the clumping phenotypic variation into the QTL gene *AMN1*. We further identified the V368D substitution in Amn1 as the causative variation of the QTL gene through site specific mutation and allele replacement experiments [11]. Amn1 was previously found to be required to turn off the mitotic exit network (MEN), a pathway that promotes spindle breakdown, degradation of mitotic cyclins, cytokinesis, and post-mitotic cell separation, through obstructing the binding of Tem1 to Cdc15 [12, 13].

This paper presents a novel mechanism of Amn1-mediated cell separation inhibition after mitosis in YL1C. We show here for the first time that Amn1 can post-translationally control the degradation of Ace2 through the ubiquitin proteasome system (UPS). This establishes that Amn1 modulates post-mitotic cell separation through down-regulating Ace2 and its downstream genes. The data not only advances Amn1 as an antagonist of MEN [12], but also provides a new insight into how the RAM mediates post-mitotic cell separation [4–7]. Moreoever, we demonstrate that the Amn1-governed post-mitotic cell separation is cell-type dependent. The clumping phenotype governed by Amn1 is highly dependent on the ploidy level in natural *S*. *cerevisiae* cells, while the functional of Amn1^368D^ from YL1C in controlling post-mitotic cell separation, is evolutionarily conserved in both genic structure and phenotypic effect.

## Results

### *AMN1* Inhibits post-mitotic cell separation and causes cell clumping

**Fig 1:**
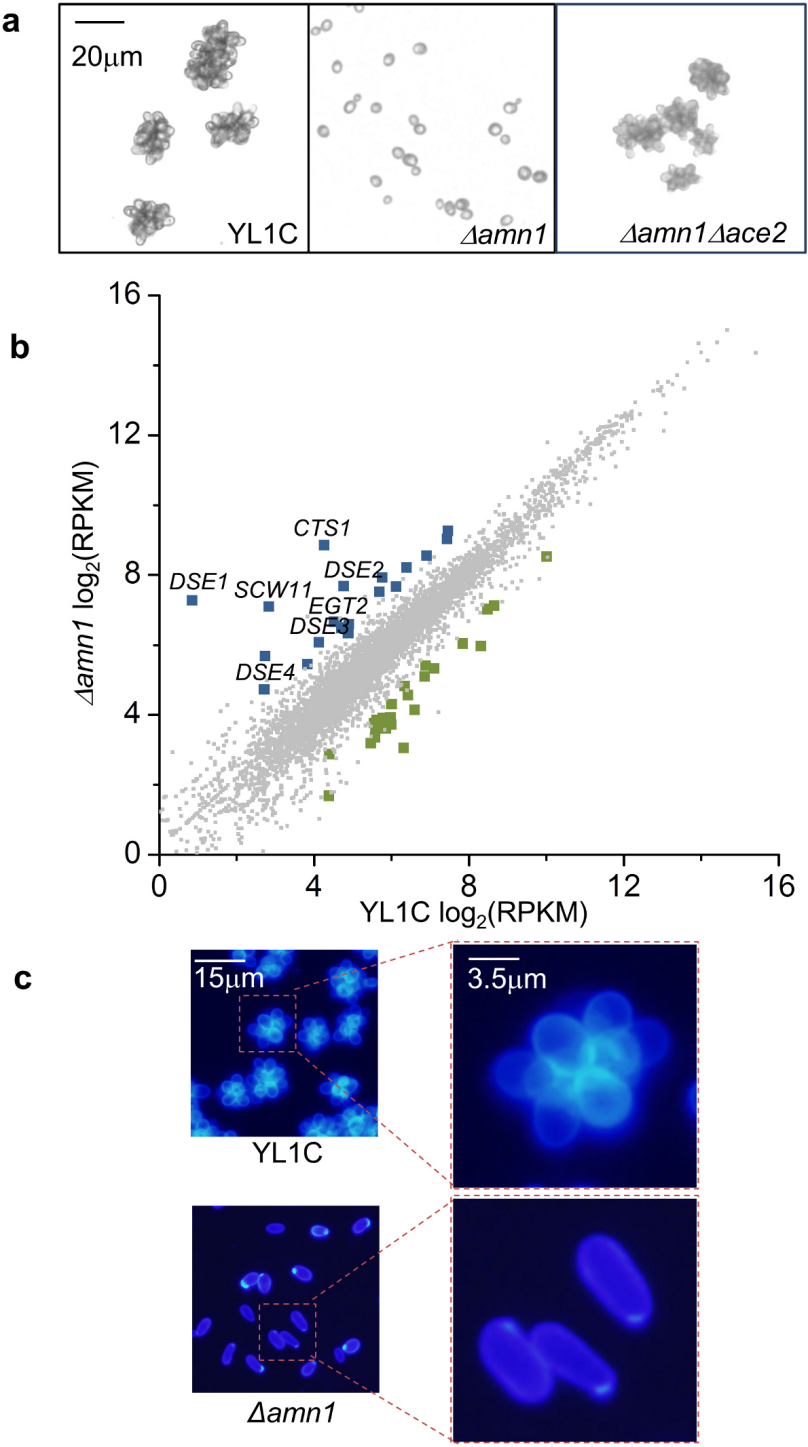
Inhibited post-mitotic cell separation in YL1C strain. **(a)** Cell clumping phenotype observed under the microscope of YL1C, the *Δamn1* mutant and *Δamn1Δace2* mutant. **(b)** Differentially expressed genes between YL1C and YL1C *Δamn1*. Compared to YL1C, up-regulated genes in the YL1C *Δamn1* mutant are labeled with blue, down-regulated genes are labeled with green, whilst genes not significantly differentially expressed between the two strains are labeled with grey. Ace2 target genes are labeled above the corresponding spots. **(c)** Images of calcofluor white stained cells of YL1C and YL1C *Δamn1*.

Firstly, the clumping cells of the *S*. *cerevisiae* strain YL1C became separated when *AMN1* was deleted (Fig 1A) as we previously observed [11]. To explore the underlying mechanism by which *AMN1* causes cell clumping, we conducted an RNA-seq assay and identified 43 significantly differentially expressed genes between YL1C cells showing a strong clumpy phenotype and YL1C with *AMN1* deleted (Fig 1B). Of these 43 genes, 18 were up-regulated when *AMN1* was deleted, including *DSE1*, *DSE2*, *DSE3*, *DSE4*, *EGT2*, *SCW11* and *CTS1* with known roles in post-mitotic cell separation, acting directly to degrade the primary septum at the bud neck [14–16]. From these 7 known genes, we chose the 4 most up-regulated genes, *DSE1*, *DSE2*, *SCW11* and *CTS1*, and confirmed the results of the RNA-seq assay by using RT-qPCR (S1 Fig). We then stained YL1C cells with calcofluor white (CFW), a fluorescent dye specifically staining chitin, the major component of the septum, as previously suggested [17], and confirmed that the YL1C cells remained attached with the undegraded primary septum at the bud neck. In contrast, when *AMN1* was deleted, the bud scars were deeply stained by CFW, indicating complete mother-daughter cell separation (Fig 1C). These results indicate that *AMN1* inhibits cell separation after mitosis and induces cell clumping as seen in the YL1C strain.

Yeast cell clumps can also be formed through an aggregate cellular behavior genetically controlled by the FLO family of genes that regulate interactions between cell wall glycoproteins [18–20]. We invesigated the influence of FLO genes on the clumping of YL1C cells. *FLO1* and *FLO8* deletion showed a comparable clumping phenotype to that of YL1C (S2 Fig), suggesting that clumping phenotype of YL1C cells was largely attributable rather to defective post-mitotic cell separation governed by Amn1 than to the cell wall glycoproteins encoded by the FLO gene family.

### Down-regulation of Ace2 by Amn1

**Fig 2:**
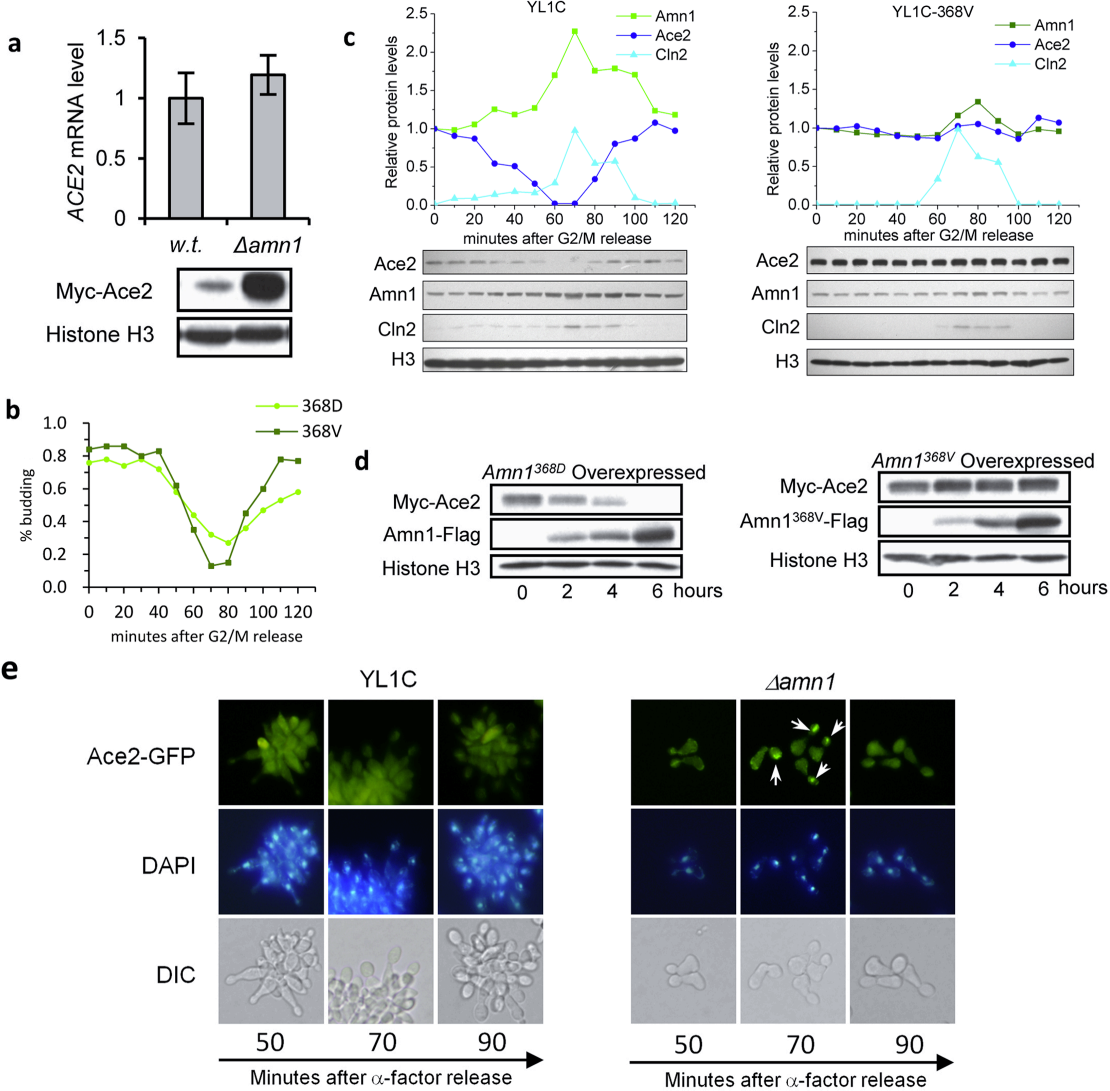
Down-regulation of Ace2 by Amn1. **(a)** RNA and Protein levels of *ACE2* in YL1C and *Δamn1* mutant. **(b)** Budding index of YL1C and YL1C-368V cells. **(c)** Protein levels of Ace2 and Amn1 in synchronized YL1C and YL1C-368V strains. Immunoblotting was performed every 10 minutes after cells were released from G2/M arrest. The bands of Ace2/Amn1/Histone H3/Cln2 were quantified using Quantity One (BioRAD) and the data was shown after normalization. Cln2 and H3 were used as a cell cycle marker and loading control, respectively. **(d)** The protein levels of Amn1^368D^ (or Amn1^368V^) and Ace2 in YL1C background strains. *AMN1* was driven by *P*_*GAL10*_, and the cells were sampled at 0, 2, 4 and 6 hours after adding galactose. **(e)** Nuclear accumulation of Ace2-GFP in YL1C and the *Δamn1* mutant. Ace2-GFP is shown in the up panel, nuclear DNA stained with DAPI (4',6-diamidino-2-phenylindole) in the middle panel, and the corresponding DIC views in the lower panel. Cells for the assay were collected and assessed at 50-, 70- and 90- minute time points after α-factor release.

Ace2 is the major transcription factor of *DSE1*, *DSE2*, *DSE3*, *DSE4*, *EGT2*, *SCW11* and *CTS1*, its mutation or deletion may sharply decrease the RNA levels of these target genes [4–6, 14–16]. Based on our observation that deletion of *AMN1* occurred in parallel with down-regulation of these Ace2 target genes (Fig 1B), and the fact that deletion of *ACE2* restored cell clumping phenotype in *Δamn1* mutant cells (Fig 1A), we hypothesized that *AMN1* inhibited post-mitotic cell separation in the YL1C strain through inactivating Ace2. To test the hypothesis, we firstly profiled the RNA level of *ACE2*, and did not find any significant change in the RNA level (Fig 2A upper), but the protein level of the gene measured by the western blotting assay was substantially up-regulated in the YL1C strain with *AMN1* deleted when compared to that in the YL1C strain (Fig 2A lower). The protein level of Ace2 was also found to vary over the cell cycle and to be negatively correlated with the protein level of Amn1 in the YL1C strain in a cell synchronization analysis by using nocodazole. However, the protein level of Ace2 did not show a marked change across the cell cycle when the cells carried an Amn1^368V^ variant (Fig 2B and 2C).

We induced expression of *AMN1* under the *P*_*GAL10*_ drive in YL1C using galactose, and profiled the protein levels of Ace2 and Amn1. The results showed that the protein level of Ace2 decreased as the protein level of Amn1 increased. At the 6-hour time point, Amn1 protein expression reached its highest level and the Ace2 protein was not detectable (Fig 2D). However, the RNA expression of *ACE2* did not show any change, while the RNA level of *AMN1* progressively increased (S3 Fig). Moreover, when the 368D was replaced by the Val residue, the protein level of Ace2 was no longer dependent on the Amn1^368V^ level (Fig 2D). The negative correlation in protein level between Ace2 and of Amn1 in both cell synchronization assay and galactose pulse-chase assay strongly support the down-regulation of Ace2 by Amn1.

We also tested the nuclear accumulation of Ace2, which was necessary to induce transcription of its target genes [4, 5]. We marked Ace2 with GFP and found that Ace2 was absent in daughter cell nuclei of synchronized YL1C cells. In contrast, when *AMN1* was deleted, Ace2 efficiently accumulated in daughter cell nuclei (Fig 2E). Using site-directed mutagenesis, we constructed two YL1C strains with a continuously activated Ace2, carrying either of two sets of multiple substitutions, either S122D, S137D, T575A, S701A and S714A (referred to as Ace2-AAA-2D), or F127V, T575A, S701A and S714A (referred to as Ace2-AAA-F127V). Both were previously reported to locate in the nucleus in all cells regardless of cell cycle position and to continuously transcribe Ace2’s target genes [5, 21]. Nevertheless, post-mitotic cell separation was inhibited in both genetically modified strains, while the RNA levels of Ace2 target genes did not significantly change in the two strains compared to YL1C (S4A Fig). Ace2 protein levels remained extremely low in the wild-type YL1C cells and its engineered strains, but were boosted when *AMN1* was deleted (S4B Fig). The data supports that Amn1 mediated degradation of Ace2 overrides the well-established view that regulation of Ace2 function is through phosphoration and protein localization during the process of cell separation after mitosis [4,5].

### Amn1 mediates turnover of Ace2 through the ubiquitin proteasome system

To further explore downregulation of Ace2 by Amn1, we firstly performed ribosome profiling to test whether Amn1 affects the translational efficiency of Ace2. It shows no marked change in the level of *ACE2* mRNAs occupied by the ribosomes in YL1C compared with the corresponding *Δamn1* strain (S5 Fig). The downregulation of Ace2 by Amn1 is therefore unlikely due to altered translational efficiency.

**Fig 3:**
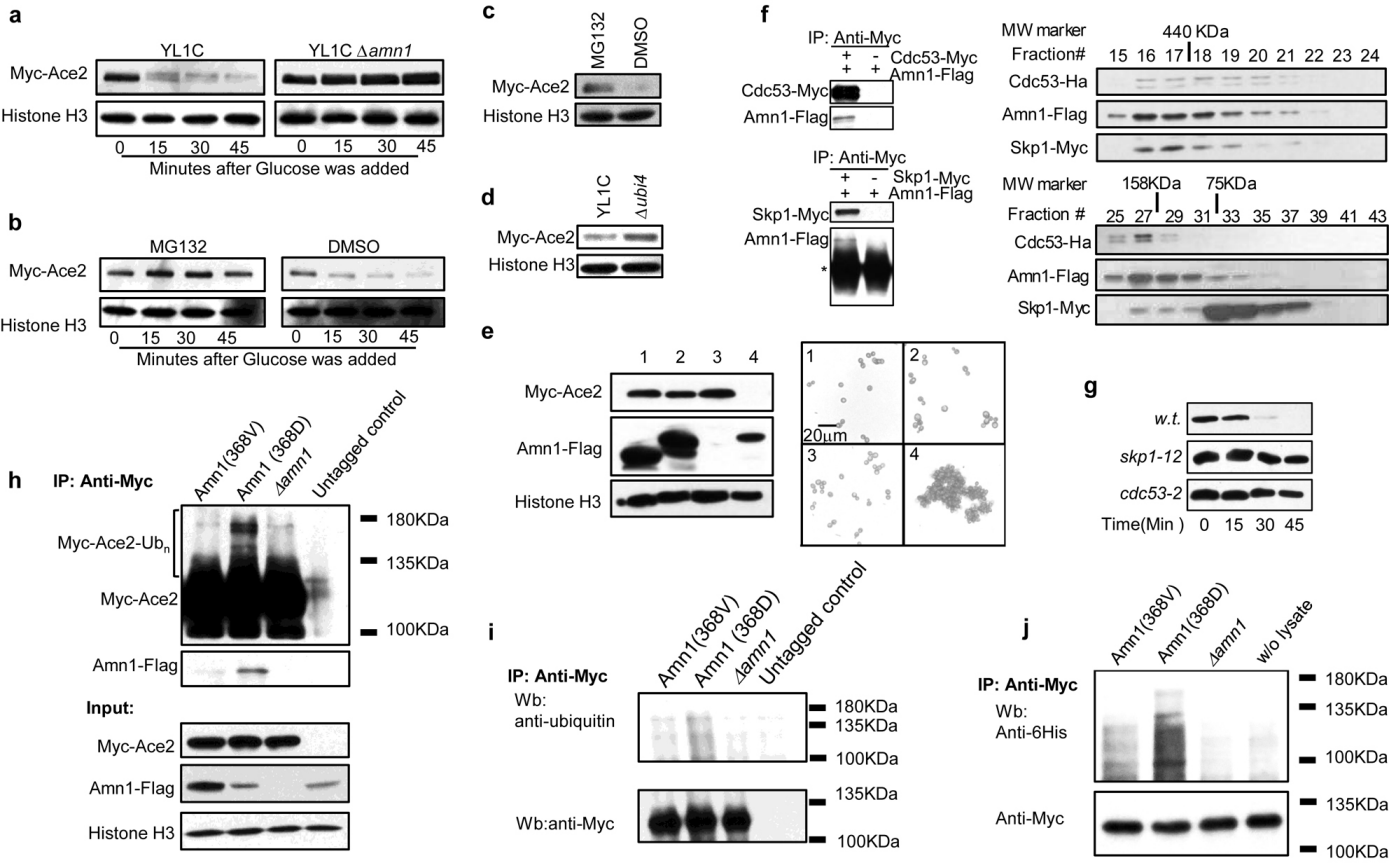
Amn1 mediates turnover of Ace2 through the UPS. **(a)** Ace2 protein levels in YL1C or YL1C *Δamn1* strain were quantified using western blotting every 15 minutes since the inducible transcription of *ACE2* by *P*_*GAL10*_ was shut down by glucose. **(b)** Detection of Ace2 protein in YL1C *Δpdr5* strain treated with MG132 after the inducible transcription of *ACE2* was turned off. DMSO was used as the control. **(c)** Endogenous Ace2 protein levels in YL1C *Δpdr5* strain treated with MG132. DMSO was used as the control. **(d)** Endogenous Ace2 protein levels in YL1C and YL1C *Δubi4* strains. **(e)** Protein level of Amn1 in YL1C with the F-box being deleted and the corresponding microscope images of cell clumping phenotype as shown below, showing YL1C with: (1) *AMN1Δ(*496–789) overexpressed, (2) *AMN1Δ(*496–552)&*(*721–789) overexpressed, (3) *AMN1* deleted and (4) *AMN1* overexpressed. **(f)** Physical interactions of Amn1 with Cdc53 and Skp1 were detected through co-IP (left panel) and size-exclusion chromatography experiment (right panel) in YL1C strains.* antibody heavy chain **(g)** Ace2 protein levels in YL1C, *skp1-12* or *cdc53-2* mutant were quantified using western blotting every 15 minutes since the inducible transcription of *ACE2* by *P*_*GAL10*_ was shut down by glucose. **(h)** In YL1C strains, Myc-Ace2 was expressed from the GAL promoter and the Ace2 protein complex enriched by use of IP and quantified by western blotting with anti-Myc for Ace2 and anti-Flag for Amn1. Ace2 and Amn1 were tagged with Myc and Flag, respectively. Columns from left to right indicate: YL1C with the *AMN1*^368V^ allele substituted *in situ*, wild-typed YL1C, YL1C *Δamn1*, and YL1C with untagged *ACE2* as the control. **(i)** In YL1C strains, the GAL promoter inducibly expressed Ace2 as described in (h) was first immuno-precipitated and then detected by western blotting by use of the anti-ubiquitin, and, in parallel, by use of anti-Myc after the antibody was stripped from membrane. The columns from the left to right: YL1C with the *AMN1*^*368V*^ allele substituted *in situ*, the wild-typed YL1C, YL1C *Δamn1*, and YL1C with untagged *ACE2* as the control. (j) *in vitro* ubiquitination assay. The cell lysate extracted from the left column to the right: YL1C with the *AMN1*^*368V*^ allele substituted *in situ*, the wild-typed YL1C, YL1C *Δamn1*, and lysis buffer (i.e. contained no cell lysate). The assay is detailed in Materials and Methods.

To examine the apparent impact of Amn1 on the stability of Ace2, we performed a GAL promoter shut-off chase experiment in which *P*_*GAL10*_*-ACE2* was transformed into YL1C so as to drive *ACE2* expression. The engineered cells were initially grown in galactose medium to induce *ACE2* expression, and then transferred to glucose medium to switch off *ACE2* transcription. The protein level of Ace2 was then measured every 15 minutes using western blotting. Ace2 was extremely unstable and vulnerable to degradation in the YL1C strain (Fig 3A left panel), while no marked decrease in the protein level was observed in the *AMN1* deleted strain (Fig 3A right panel). When the proteolytic activity of the 26S proteasome was blocked using MG132, then Ace2 levels no longer markedly decreased (Fig 3B). Additionally, YL1C cells treated with MG132 had a stable endogenous Ace2 protein level (Fig 3C), while the endogenous Ace2 protein was markedly boosted when the ubiquitin coding gene *UBI4* was deleted (Fig 3D). These results indicated that the down-regulation of Ace2 by Amn1 can be explained by the ubiquitin-conjugated protein degradation machinery.

Amn1 was previously predicted to be a member of the F-box protein family in *S*. *cerevisiae*, and proposed to be a potential ubiquitin ligase E3 in a bioinformatic analysis [22]. However, the putative F-box in Amn1 is atypical since the motif is separated by a 56-amino acid insertion. However, there is no experimental evidence so far for whether Amn1 catalyzes ubiquitination of Ace2 directly and facilitates its degradation. To tackle this open question, we firstly constructed two genetic modified Amn1 proteins without a normal function as an E3 ligase, one with deletion of only the F-box (referred to as *AMN1-Δ*(*496–552*)*&*(*721–789*)) and the other with deletion of both the F-box and the 56 amino acid insertion (referred to as *AMN1-Δ*(*496–789*)). YL1C cells with either modified Amn1 protein showed effective cell separation and substantial up-regulation of the Ace2 protein *in vivo* (Fig 3E). This suggested that the atypical F-box in Amn1 was functional and controlled the stability of Ace2.

Furthermore, we carried out co-immunoprecipitation assays and showed that Amn1 physically binds to Skp1 and Cdc53, two key components of SCF complex (Fig 3F left panel)[23]. We also performed a size-exclusion chromatography experiment, and found that the three proteins, Amn1-Flag, Skp1-Myc and Cdc53-Ha, could be co-eluted at #16-#21, corresponding to the apparent size of ~440kDa. Additionally, we found that the three proteins overlapped again at fraction #27-#29, corresponding to the apparent size of ~ 158kDa. We thus speculated that the fraction #27-#29 contained the complexes of Amn1-Cdc53, Cdc53- Skp1, and Amn1-Skp1 (Fig 3F right panel). We then examined the stability of Ace2 in *cdc53* and *skp1* mutants using the GAL promoter shut off assay. And found Ace2 was greatly stabilized by mutations in both *CDC53* and *SKP1* (Fig 3G). Moreover, we enriched the Ace2 protein complex using immunoprecipitation and profiled Amn1 and Ace2. Amn1 in YL1C was clearly seen to physically interact with Ace2 (Fig 3H). However, when the 368D in Amn1 was substituted by Val, the Amn1^368V^ level was detected but its physical interaction with Ace2 was substantially weakened. A higher molecular ladder of ubiquitinated Ace2 was also detected in YL1C (Fig 3H and 3I), while ubiquitinated Ace2 was almost completely eliminated (Fig 3H and 3I) when *AMN1* was deleted or substituted with *AMN1*^368V^. An *in vitro* protein ubiquitination assay also showed that Ace2 could be labelled with exogenous human ubiquitin in the presence of Amn1^368D^, but not in the absence of Amn1, and the ubiquitination was substantially weakened when Amn1^368D^ was replaced by Amn1^368V^ (Fig 3J). These observations support Amn1’s role in mediating Ace2 proteolysis through the UPS, and the essentiality of the 368D residue to enable Amn1 to adequately bind and hence to ubiquitinate Ace2.

### A 11-residue domain of Ace2 as the binding target for Amn1 to mediate Ace2 proteolysis

**Fig 4:**
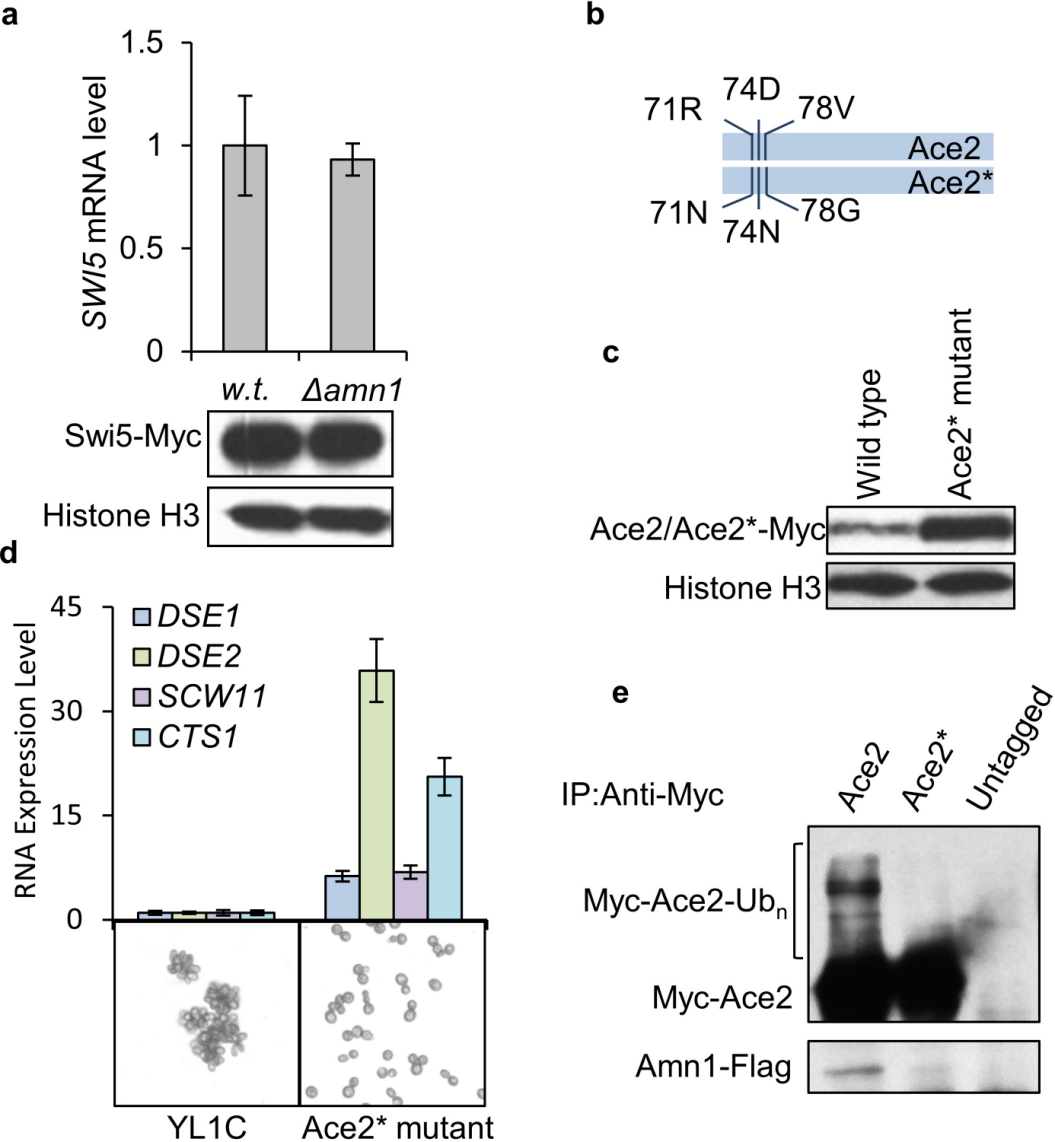
A 11-residue domain of Ace2 as the binding target for Amn1 to mediate Ace2 proteolysis. **(a)** RNA (by RT-qPCR assay) and protein (by western blotting assay) levels of *SWI5* in YL1C and YL1C *Δamn1* strains. **(b)** the diagram of Ace2*(where the 11-residue domain of Ace2 was replaced by the corresponding aligned region in Swi5). **(c)** Protein levels of Ace2 or Ace2* under YL1C background with an endogenous promoter of *ACE2*. **(d)** Microscope images of cell clumping phenotype of YL1C and Ace2* mutant. The corresponding RNA levels of *DSE1*, *DSE2*, *SCW11* and *CTS1* were presented above the images. **(e)** In YL1C/Ace2* mutant strains, Ace2 and Ace2* protein complexes were respectively captured by anti-Myc and detected by western blotting with anti-Myc for Ace2 and anti-Flag for Amn1. Ace2 and Amn1 were tagged with Myc and Flag, respectively. Untagged Ace2 was used as the IP control.

*ACE2* has a paralog *SWI5* which arose from whole genome duplication [24]. Swi5 has an almost identical DNA-binding domain to Ace2, but regulates a different set of genes *in vivo* [16]. However, we did not see any significant change in RNA or protein levels of *SWI5* in YL1C with or without *AMN1*, clearly indicating that Swi5, unlike Ace2, is not regulated by *AMN1* (Fig 4A). We aligned protein sequences of Ace2 and Swi5, compared their functional domains and identified 3 aligned regions (A, B and C), with region A subdivided into A1, A2, A3 and A4 regions. We then constructed a series of Ace2-Swi5 chimeras, and identified a novel 11-residue domain ‘ELRDLDIPLVP’ as the site for Amn1 to bind to and directly degrade Ace2 (see Supplementary Materials and Methods, S6 Fig). We replaced this domain with the corresponding Swi5 derived ‘EINDLNLPLGP’ in YL1C, creating a modified Ace2* (Fig 4B). YL1C cells carrying the Ace2* separated effectively and a western blotting assay indicates that the modified Ace2* was expressed at markedly higher levels than the wild-typed Ace2 *in vivo* (Fig 4C and 4D). In addition, the RNA levels of four Ace2 target genes were clearly up-regulated (Fig 4D). An Ace2 protein with the 11 residue domain mutated may therefore escape from the negative control by Amn1. Moreover, we did not observe any detectable signal of interaction of Ace2* with Amn1, nor its ubiquitination, further supporting the role of the identified 11-residue domain in the post-translational regulation of Ace2 (Fig 4E). We then constructed a series of strains bearing single amino acid substitutions (Ace2^R71N^, Ace2^D74N^ and Ace2^V78G^), but none of these led to effective cell separation, suggesting that the whole 11-residue domain be essential for its phenotypic effect (S6C Fig).

### The Amn1-mediated post-mitotic cell separation phenotype is highly cell type dependent

**Fig 5:**
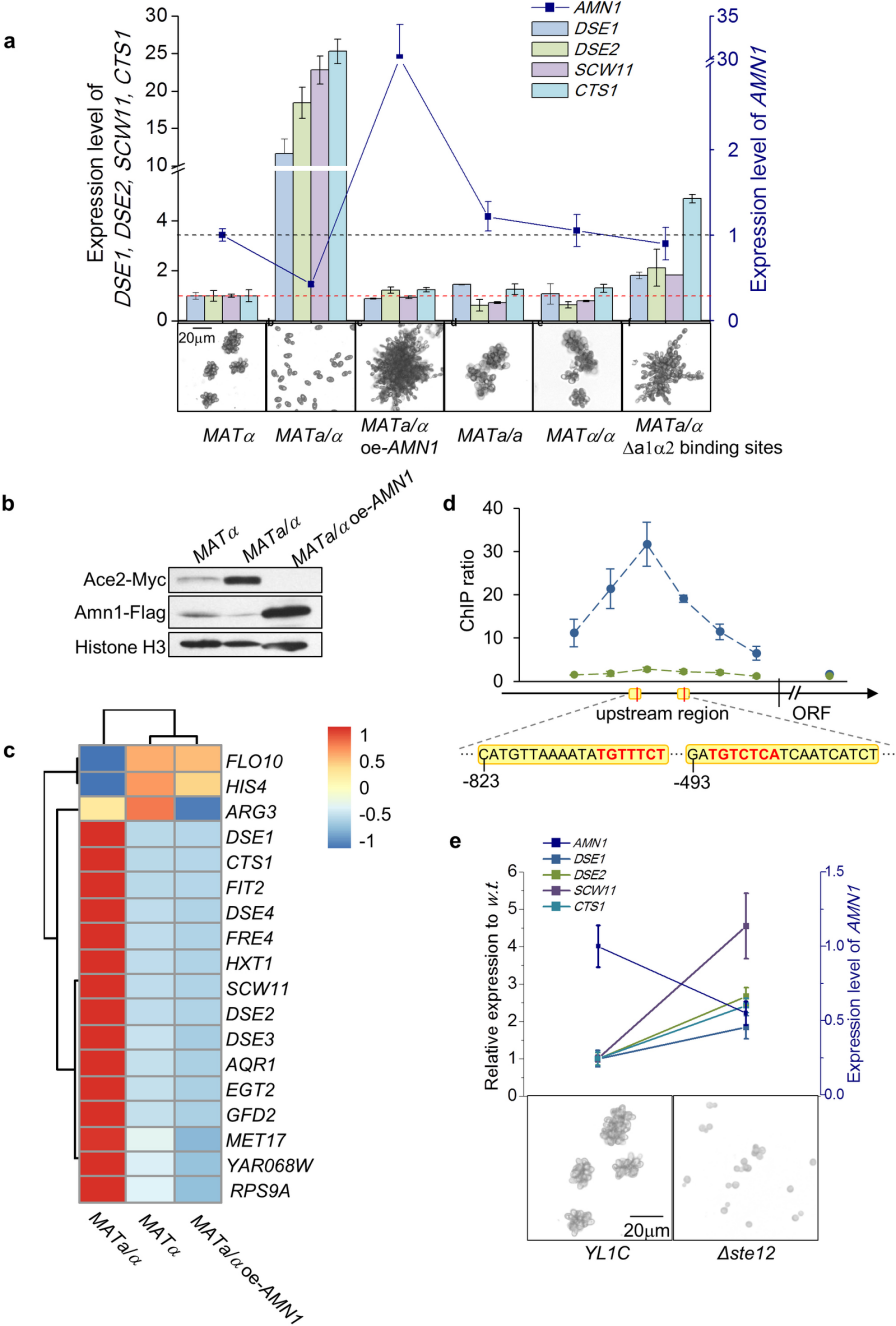
Cell type dependent post-mitotic cell separation mediated by Amn1. **(a)** RNA levels of *AMN1* and *DSE1*, *DSE2*, *SCW11* and *CTS1* in, from left to right, YL1C haploids, YL1C diploids, diploids with *AMN1* overexpressed, diploids with mating type *MATa/a* or *MATα/α*, and diploids with the binding sites of the a1-α2 heterodimer in the *AMN1* promoter deleted. Shown below are the corresponding microscope views for clumping phenotypes of the tested strains. **(b)**Protein levels of Ace2 and Amn1 in YL1C haploids, diploids or diploids with *AMN1* overexpressed. **(c)**Heatmap showing RNA levels of the 18 up-regulated genes (identified when *AMN1* was deleted) in YL1C haploids, diploids or diploids with *AMN1* overexpressed. **(d)** Ste12 ChIP-qPCR assays for haploid (blue dots) and diploid (green dots) YL1C. Illustrated on the X-axis is a part of the *AMN1* upstream region and the tail of the *AMN1* ORF, which was used as a control in the ChIP experiment. Binding sites of Ste12 (shown in red letters) and a1-α2 heterodimer (shown with a yellow background) are shown below the X-axis. The Y-axis shows the fold change of ChIP enrichment. **(e)** RNA levels of *AMN1* and *DSE1*, *DSE2*, *SCW11* and *CTS1* in the YL1C and *Δste12* mutant (*Δste12*). Shown below are microscope views of the cell clumping phenotype.

The above analyses were established in haploid cells. We found that inhibition of post-mitotic cell separation was released and the cells separated effectively in diploids (Fig 5A). Additionally, overexpression of *AMN1* led to strong inhibition of post-mitotic cell separation in the diploid cells (Fig 5A). We then checked the protein level of Amn1 and Ace2 in diploid cells using western blotting and found Amn1 protein level was much lower, agreeing with its RNA level (Fig 5A and 5B). Conversely, the protein level of Ace2 was markedly boosted in diploids compared to haploid cells (Fig 5B). Overexpression of *AMN1* in the diploid cells depressed the Ace2 protein level, as expected (Fig 5B). Using RNA-seq, we surveyed the RNA levels of the 18 genes that were up-regulated when *AMN1* was deleted in haploid cells. Of the 18, 15 showed an inflated expression in *MATa/α* diploid cells, but their RNA levels were repressed when the cells had *AMN1* over-expressed (Fig 5C). These results were confirmed by RT-qPCR assays (Fig 5A). The data supports Amn1 as the key inhibitor of post-mitotic cell separation in diploid as well as in haploid cells, but it must be noted that its endogenous expression is not activated in diploid cells in nature as to be explained below.

Diploid *S*. *cerevisiae* cells differ from the corresponding haploids in two ways. First, haploid cells and diploid cells created from merging of the haploid cells may be identical at every gene in the genome except at the *MAT* locus. Second, they differ in gene dosage [25]. To compare the diploids and haploids in exactly the same genetic background, we created diploids with a homozygous genotype *MATa/a* or *MATα/α* at the *MAT* locus. We observed that the cell clumping phenotype as well as the expression patterns of *AMN1* and its downstream regulated genes in these diploids were comparable to those in the haploids (Fig 5A). These observations effectively excluded the possibility that differentiation in the post-mitotic cell separation between natural haploid and diploid cells was due to the difference in gene dosage.

We next explored the difference in post-mitotic cell separation behavior between diploid cells with either homozygous or heterozygous genotypes at the *MAT* locus. First, we noted that the *MATa/α* diploid cells of *S*. *cerevisiae* encoded a heterodimer, a1-*α*2, which played a role as a transcriptional repressor of haploid-specific genes [26] and might contribute to ploidy specific phenotypes observed between natural haploid and diploid yeasts [26–28]. To establish association of the a1-*α*2 heterodimer with the Amn1-regulated post mitotic cell separation, we notified that the binding sites of the heterodimer in the upstream promoter region of *AMN1* predicted from publicly available large scale ChIP data [26,29]. The two predicted binding sites were also shared by another regulator, Ste12 (Fig 5D). At the two a1-*α*2 binding sites, we constructed single-site and double-site deletion mutants of the *MATa/α* diploid cells, and observed that, the *MATa/α* diploid cells in which both binding sites were deleted displayed inhibited mother-daughter separation after mitosis, similar to that observed in the corresponding *a/a* or *α/α* diploid cells (Fig 5A). At the same time, the expression of *AMN1* was markedly boosted, while the expression of Ace2’s downstream genes, *DSE1*, *DSE2*, *SCW11* and *CTS1*, was reduced (Fig 5A). We thus hypothesized that the post-mitotic cell separation of the natural *MATa/α* diploid cells was caused by the a1-α2 heterodimer blocking the binding of Ste12 to the *AMN1* promoter.

We carried out a ChIP assay to test if Ste12 binds to the *AMN1* promoter in YL1C haploids and diploids. The assay showed that the fold change of enrichment was highly significant in the haploid cells, with the enrichment peak observed in a region from -600 to -700 bp spanning the upstream region of *AMN1*, while no enrichment was observed in the diploid cells (Fig 5D). Consistently, the RNA level of *AMN1* was reduced when *STE12* was deleted in haploid cells, and in turn, the depressed expression of Amn1 has led to a remarkable boost in expression of *DSE1*, *DSE2*, *SCW11* and *CTS1* (Fig 5E). These results show that Ste12 and the a1-α2 heterodimer share common binding sites in the promoter of *AMN1*. The a1-α2 heterodimer down-regulates Amn1 and make the diploid cells effectively separate after mitosis.

**Fig 6:**
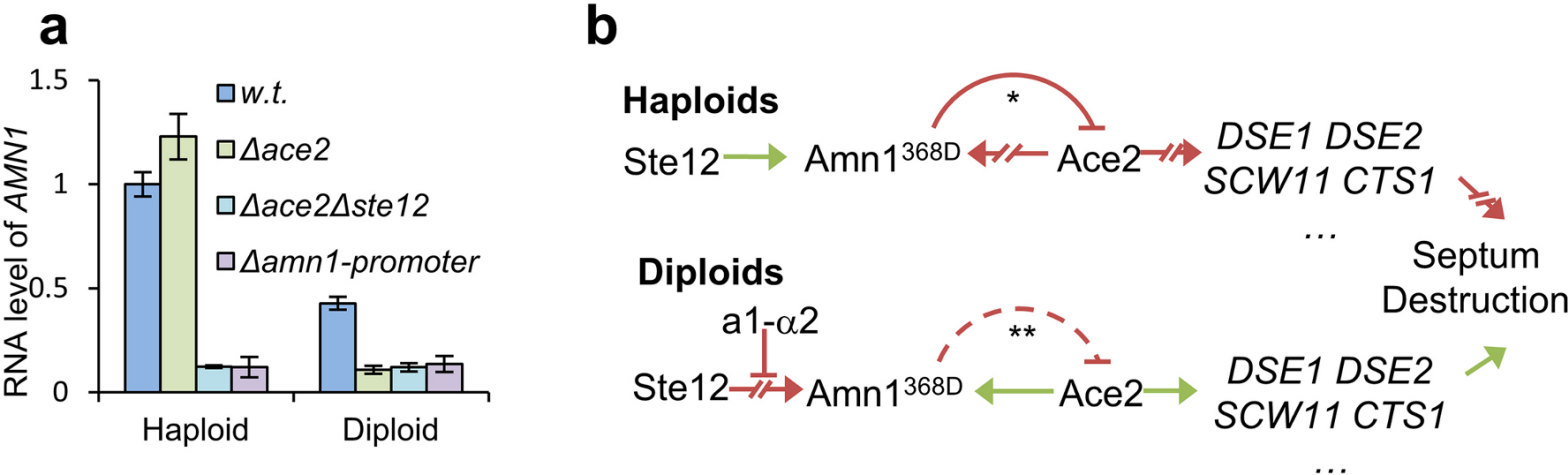
A schematic model for cell type dependent *AMN1* regulated cell separation. **(a)** RNA levels of *AMN1* in the YL1C, *Δace2*, *Δace2 Δste12* mutant and *AMN1* promoter deleted mutant in both haploid and diploid strains. **(b)** Green arrows indicate “enact or promote”, and red lines with a blunt end represent down-regulation of RNA or protein levels. Broken red arrows show potential activation pathways that have been repressed. In haploid YL1C cells that have no a1-α2 heterodimer, *AMN1* is activated by Ste12 and then degrades the transcription factor Ace2 through the UPS, and in turn, suppresses expression of Ace2’s target genes. It should be noted that Ace2 is also the constituent transcriptional factor of *AMN1* but it will not activate expression of *AMN1* in haploid cells as illustrated by the broken red arrows. That is, *AMN1*’s expression is actually regulated by Ste12 in haploids. On the other hand, in diploid YL1C cells, the a1-α2 heterodimer may inhibit Ste12’s binding to *AMN1*’s upstream region, thus Ste12’s regulation of *AMN1* will be greatly weakened. Ace2 proteins thus accumulate and activate the target genes including *AMN1*. *In haploid YL1C cells, Amn1 activated by Ste12 mediates turnover of Ace2 through the UPS. ** Whether Amn1 activated by Ace2 will form a negative feedback loop that degrades Ace2 in diploids remains to be clarified.

Although Ace2 was a well-documented transcription factor of *AMN1* in S288C or W303 yeasts, Ace2 was absent in haploid YL1C [16]. Thus, the question rises how *AMN1* is regulated in YL1C? We showed that the RNA level of *AMN1* in haploid cells with *ACE2* deleted was not reduced to the background level (i.e. the level when *AMN1*’s promoter was completely removed) until *STE12* was additionally deleted. In contrast, a single deletion of *ACE2* was sufficient to reduce the RNA level of *AMN1* to the background in the diploid cells (Fig 6A). All these together show that transcription regulation of *AMN1* in YL1C cells differs between the two types (haploid and diploid) of cells.

In summary, the heterodimer is absent in haploids and Ste12 activates *AMN1*. Expression of *AMN1* silences the protein expression of Ace2 through its N-terminal 11-residue domain as illustrated in Fig 4 and thus inhibits expression of Ace2 target genes. Down-regulation of the genes involved in septum cleavage consequently leads to post-mitotic cell separation inhibition, causing cells to be clumped in haploids. On the other hand, in *MATa/α* diploid strains, the presence of the a1-α2 heterodimer prevented Ste12 from efficiently binding to the *AMN1* promoter, which in turn, inactivates the transcription of *AMN1*. This leads to effective post-mitotic cell separation because Ace2 is then released from negative regulation by Amn1. These observations explain the cell type dependency of the Amn1 regulated cell separation after mitosis (Fig 6B).

### Evolutionary conservation of Amn1^368D^ intra and inter yeast species

All the conclusions above were drawn from experiments with natural, cell clumped strain YL1C, which carrying an *AMN1*^*368D*^. To explore the conservation of Amn1^368D^ among yeast strains of *Saccharomyces cerevisiae*, we collected and aligned the Amn1 protein sequences for all 46 sequenced yeast strains from the SGD database (www.yeastgenome.org). Of the 46 strains, 34 shared the 368D in Amn1, while the remaining 12, most of which have a common S288C background, (e.g. S288C, W303, BY4741, BY4742, JK9-3d and X2180-1A), carry the 368V (S2 Table). These data prompt us that *AMN1*^*368D*^ is a dominant allele, while *AMN1*^*368V*^ probably sourced from the human-selection for a lab-used strain which was less clumpy and thereby greatly easily handled.

**Fig 7:**
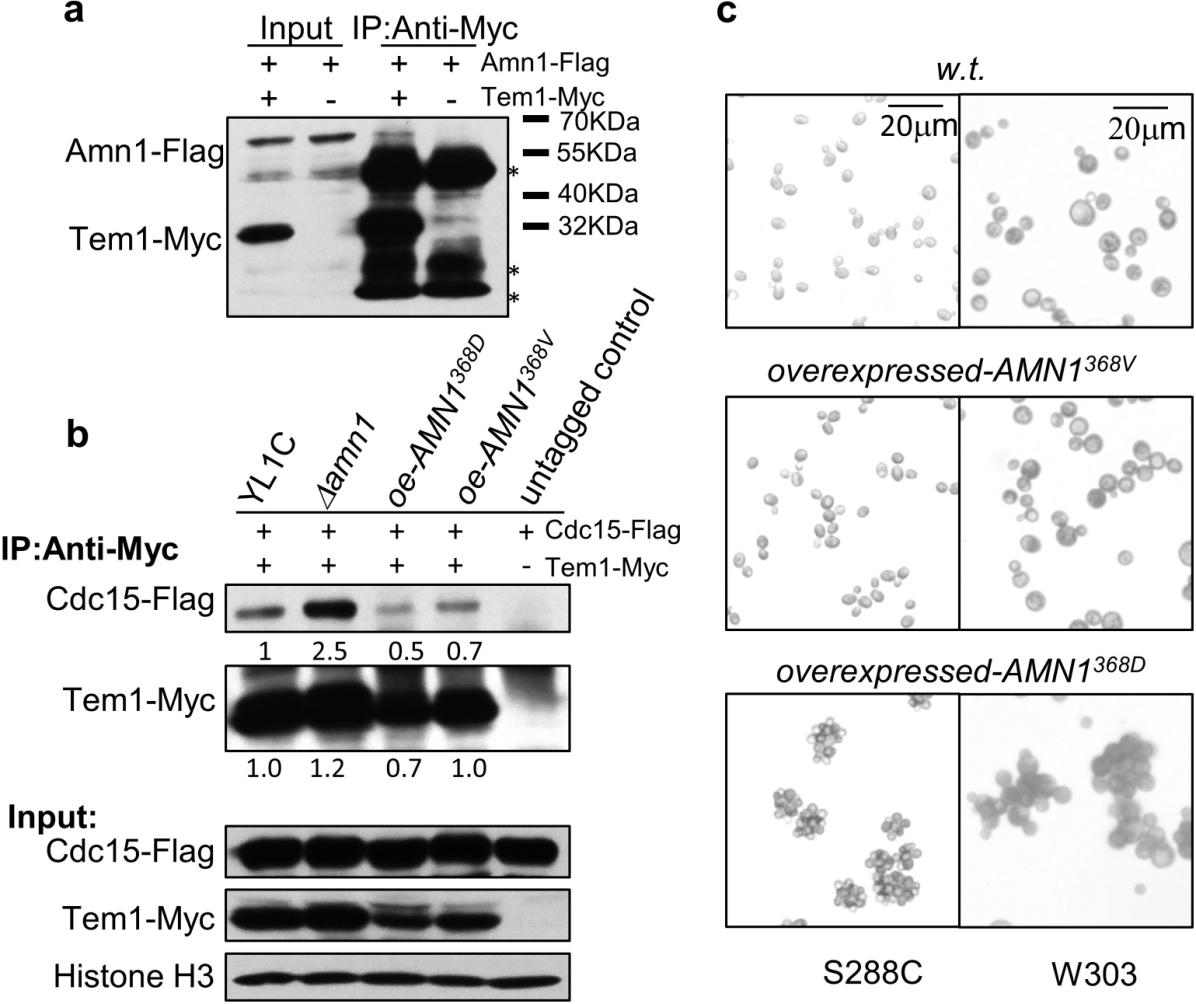
Evolutionary conservation of Amn1^368D^ in post-mitotic cell separation. **(a)** Physical interaction between Amn1^368D^ and Tem1 was detected by co-IP in YL1C. * antibody heavy/light chains or unspecific bands. **(b)** co-IP assays in YL1C, YL1C *Δamn1*, and YL1C with overexpressed Amn1^368D^ or Amn1^368V^ for detecting physical interaction between Cdc15 and Tem1. The bands were quantified using Quantity One (BioRAD) and the data was shown after normalization. **(c)** Microscope views of cell clumping phenotype of W303 and S288C strains or these strains with overexpressed Amn1^368D^ or Amn1^368V^ respectively.

Amn1 was reported as an inhibitor of the mitotic exit network (MEN), through binding to Tem1, and in doing so, inhibiting Tem1 binding to Cdc15 [12,13]. However, these findings were established from the haploid strain W303 carrying Amn1^368V^. To investigate whether MEN would also be inhibited in the YL1C haploid strain carrying Amn1^368D^, we performed a co-immunoprecipitation assay and observed effective binding of Amn1^368D^ to Tem1 (Fig 7A). When *AMN1* was deleted in YL1C, the interaction between Tem1 and Cdc15 was strengthened; however, once either *AMN1*^*368D*^ or *AMN1*^*368V*^ was overexpressed in the YL1C *Δamn1* cells, the interaction between Cdc15 and Tem1 was markedly weakened (Fig 7B). These observations show that Amn1^368D^ has the same function as Amn1^368V^ in suppressing the interaction of Tem1 with Cdc15, suggesting that both proteins can inhibit MEN. We also compared Amn1^368D^ and Amn1^368V^ for their role in regulating post-mitotic cell separation in both S288C and W303 strains. The comparison shows that overexpressed *AMN1*^*368D*^ may still result in post-mitotic cell separation inhibition, whereas overexpression of *AMN1*^*368V*^ does not lead to the same phenotype in these two strains (Fig 7C).

*AMN1* was predicted as an orthologous gene among at least 10 yeast species from the orthologous groups database (OrthoDB, http://orthodb.org/orthodb7), including *S*. *cerevisiae*, *C*. *glabrata* and *K*. *lactis* [30]. Notably, the conserved regions of the Amn1 orthologous proteins cover the functional 368D residue (S7A Fig). We tested functional conservation of the orthologous proteins in two yeast species related to *S*. *cerevisiae*, diverging ~100 million years ago [31]. First, *C*. *glabrata*, a highly opportunistic pathogen is usually found in the urogenital tract or bloodstream and especially prevalent in HIV positive or elderly populations [32]. Second, *K*. *lactis*, a *Kluyveromyces* yeast is commonly used in basic research and industry [33]. We aligned Amn1^*Kl*^ (from *K*. *lactis*) and Amn1^*Cg*^ (from *C*. *glabrata*) with Amn1^*Sc*^ (from *S*. *cerevisiae*) using clustal W and found 24.8% and 37.6% amino acid sequence identity with Amn1^*Sc*^ respectively (S7D Fig). When we replaced the coding region of the *AMN1* allele *in situ* in YL1C with the coding region of either *AMN1*^*Cg*^ or *AMN1*^*Kl*^, the clumping phenotype was restored in *Δamn1* mutant cells (S7B Fig). The RNA levels of the four major downstream genes responsible for septum degradation, *DSE1*, *DSE2*, *SCW11* and *CTS1*, were consistently repressed in the two gene replacement strains (S7C Fig). These results indicate that *AMN1*^*368D*^, but not *AMN1*^368V^ is more likely the conserved allele.

## Discussion

The evolution of multicellularity from unicellularity in eukaryotes has attracted wide interest in both basic research and its translational value in medicine and industry. However, the underlying molecular mechanism that governs this important transition in cellular behavior is far from well established. In model organism budding yeast, the defective post-mitotic mother and daughter cell separation has been recognized as a key step in the evolution of multicellularity in the recent literature. In these works, clumped yeast cells from inhibited mother-daughter cell separation during mitosis were attributed to various mutants of *ace2*, particularly a truncated *ace2* mutant [8–10], or the inhibition of Ace2’s nuclear importation in budding yeast [4, 5]. Here, we report a novel Amn1 governed post-mitotic cell separation and demonstrate that Amn1 induces proteolysis of Ace2 through the ubiquitin proteasome system, and in turn, down-regulates Ace2’s downstream target genes involved in hydrolysis of the primary septum. This leads to inhibition of cell separation and clumping of haploid yeast cells.

Moreover, the present study reveals that separation of mother and daughter cells after mitosis regulated by Amn1 is also highly dependent on cell type in the budding yeast *S*. *cerevisiae*, with effective separation after mitosis observed when haploid cells merge with other haploid cells of an opposite mating type to form diploids. In light of the Amn1 governed post-mitotic cell separation and our observation that *AMN1* has an unusually lengthy upstream region, we demonstrate that Ste12 and the a1-α2 heterodimer share the same binding sites in the promoter of *AMN1*, which leads to the transcription regulation of *AMN1* in YL1C cells differs between the two types (haploid and diploid) of cells. The present study clarified that divergence in the regulation of post-mitotic cell separation between haploids and diploids is not attributable to gene dosage, but actually to the presence or absence of the a1-α2 heterodimer. Furthermore, up to 65 other regulators that conditionally activate *AMN1* were proposed to target the upstream region of *AMN1*, which may change the transcription regulation of *AMN1* [34]. Among them, Phd1 in diploid yeast cells may bind to the *AMN1* promoter when the cells are starved of nitrogen [35]. These observations indicate that *AMN1* could be activated conditionally by common regulators, leading to inhibition of post-mitotic cell separation.

The functionality of *AMN1* in regulating post-mitotic cell separation is highly conserved among *S*. *cerevisiae* and its two closely related yeast species, *K*. *lactis* and *C*. *glabrata*. Amn1 has not only preserved a high level of amino acid sequence similarity among yeast species, but also conservation of the functional domain of the C terminus can be extended to at least 13 other species, including *Homo sapiens*, *Xenopus tropicalis* and *Danio rerio* [36]. therefore, Amn1 may also be able to mediate turnover of substrates through the UPS in higher species, and regulate other biological processes in addition to post-mitotic cell separation.

## Material and methods

### Yeast strains and plasmid construction

All yeast strains and plasmids used in this research are listed in S3 and S4 Tables, and the methods for genetic modification of yeast strains and plasmid construction are detailed in supplementary material and methods.

### RNA-seq and RT-qPCR analysis

Yeast cells were cultured in 50 ml YPD liquid medium until OD600 = 1.0 was reached. Cells were then harvested by centrifugation and the cell pellet was washed with ice-cold water. Total RNA was extracted with the Qiagen RNeasy mini kit and then RNA-seq libraries were constructed using the Illumina TruSeq kit. RNA was quantified using the NanoDrop and RNA integrity was assessed using the Agilent BioAnalyzer 2100. Using the Illumina HiSeq 2000, about 5 million 125 bp paired end reads were collected per sample. Reads were mapped to the S288C genome for expression analysis using tophat [37]. Gene expression was measured as reads mapped per kilobase of exon per million reads mapped (RPKM). A list of differentially expressed genes was obtained by using cufflinks and cuffdiff [37]. Total RNA for RT-qPCR was isolated using the hot phenol protocol [38] followed by purification with RNase-free DNase (Promega, USA), and finally subjected to first-strand cDNA synthesis using SuperScript III Reverse transcriptase (Invitrogen, USA). 1μl of the single-strand cDNA in 10-fold dilution was used as the template for real-time quantitative PCR (RT-qPCR) using SYBR-green (Toyobo, Japan) and *ACT1* as the internal control. Every tested strain was independently cultured three times to gain three independent biologically replicated samples and each sample was assayed in triplicate in the qPCR assay.

### *AMN1* orthologs replacement experiments

*AMN1*^*Kl*^ (XM_451967.1) was cloned from genomic DNA of *K*.*lactis* (NRRL Y-1140) using primers ATGTCTTGCGTCTCCAGTATTAG and TCAGGTCGCTTCGTTGAC, whilst *AMN1*^*Cg*^ (XM_447123.1) was cloned from genomic DNA of *C*. *glabrata* (CBS 138) using primers ATGGTATTGCCTGATTCCAAC and TTATTCATTTTCTAATTGATTAAC. We used *UAR3* pop-in/pop-out method to perform *AMN1* ortholog replacement experiments. The *AMN1* ORF in YL1C was firstly deleted using the *URA3* marker, and then the *URA3* marker was replaced with *AMN1*^*KL*^ and *AMN1*^*CG*^ separately.

### Western blotting

Proteins were extracted using the protocol described by Kushnirov [39]. Briefly, 10^7^ yeast cells were harvested by centrifugation and the pellet was washed with H_2_O before incubation in 200μl 0.2M NaOH for 5 minutes at room temperature. Cells were centrifuged again and the pellet was incubated in 50μl SDS-loading buffer (120mM Tris-Cl, 10% glycerol, 4% SDS, 8% β-mercaptoethanol, 0.005% bromophenol blue), and boiled for 5 minutes. Cells were centrifuged and 10μl supernatant was loaded and separated in SDS-PAGE. Western blotting was performed using the standard laboratory procedures [40].

### Cell-cycle synchronization

Cell-cycle was synchronized by using of α–factor or nocodazole, as described previously [41]. Briefly, mating type of YL1C cells which were to be arrested with α–factor was switched to *MATa* using plasmid *pTetra*, and their *BAR1* gene was deleted using *natMX4* so that the strain could respond to a low density of α–factor. The logarithmic phase cells (OD600 = 0.5) were grown in YPD with added α–factor (50 ng/ml) for 1.5 hours. The G1-arrested cells were subsequently released after repeatedly washing with pre-warmed ddH_2_O at 30°C. The cells were then incubated in pre-warmed YPD medium with Pronase E (0.1 mg/ml). Similarly, the nocodazole-arrested assay was performed by adding 200 μl of nocodazole (1.5M) into 20 ml of logarithmic phase cells (OD600 = 0.5). The cells were grown for another 2.5 hours to be arrested in G2/M phase and then washed twice using pre-warmed YPD. The arrested cells were released in 40 ml fresh YPD medium. Finally, 2 ml samples of the cells treated either with α–factor or with nocodazole were harvested every 10 minutes over the next two hours for further analysis.

### Size exclusion chromatography

Yeast cell cultures in a volume of 50 ml were grown in YPD to an optical density of OD600 = 1.0. The cells were then harvested by centrifugation and the cell pellet was washed with ice-cold water. The cells were pelleted and re-suspended in 1000 μl of lysis buffer (50 mM HEPES-KOH at pH 7.5, 140 mM NaCl, 1 mM EDTA, 1% Triton X-100, 0.1% Na-deoxycholate, 1 tablet of the complete inhibitor cocktail supplied by Roche) and lysed with acid-washed glass beads for 15 minutes in a vortex on full output. Cell lysate was centrifuged (15 min at 13,000 rpm) at 4°C to remove cell debris. The gel filtration column (Superdex 200; Amersham Biosciences) was washed and equilibrated using cold PBS (4°C). Lysates were passed over the gel filtration column with a flow rate of 0.5 ml/min. Samples were collected every 0.5 ml per tube and analyzed by western blot.

### Immuno-precipitation

Co-immuno-precipitation assay was performed for detecting physical interaction between Ace2 and Amn1. Cells carrying pGU-Myc-Ace2 were grown in SC-U medium overnight. Cell cultures were diluted to an OD600 of 0.2 using 20ml YPR and grown to stationary phase. Cells were then harvested by centrifugation. The cell pellets were resuspended in fresh 50ml YPR and continuously cultured until OD600 reached an approximate value of 0.8. Galactose was immediately added and MG132 (20 μM in final solution) was added two hours later into the cell culture. The cells were grown for another hour and then harvested by centrifugation. WCEs (whole cell extracts) were extracted using glass beads in IP lysis buffer (50 mM HEPES-KOH at pH 7.5, 140 mM NaCl, 1 mM EDTA, 1% Triton X-100, 1 tablet of the complete inhibitor cocktail supplied by Roche) and quantified using NanoDrop. 5mg of WCEs was used as input and 50 mg (1000 μl) of WCEs were then added with magnetic dynabead protein G (Invitrogen, USA), which was pre-incubated with the monoclonal mouse anti-Myc (Transgene, China) antibody for 30 min at room temperature. The mixture was rotated for 1 hour at room temperature. The dynabeads conjugating protein complex was harvested by magnetic force and washed three times with IP lysis buffer. The beads were resuspended in SDS-loading buffer (120mM Tris-Cl, 10% glycerol, 4% SDS, 8% β-mercaptoethanol, 0.005% bromophenol blue) and boiled for 5 minutes. All samples were followed by western blotting, using anti-Myc antibody (Transgene, China) and anti-Flag (Sigma, USA). To detect the physical interactions of Cdc53/Amn1, Skp1/Amn1, Amn1/Tem1 and Cdc15/Tem1, yeast cells were grown in YPD until OD600 of 1.0. The following steps were performed as above.

### *in vitro* ubiquitination assays

*in vitro* ubiquitination assays were performed as previously described [42]. Briefly, YL1C cells expressing *AMN1*^368D^, *AMN1*^368V^ or *Δamn1* mutant cells were harvested when the OD_600_ reached 1.0. Cell pellets were resuspended in reaction buffer (50 mM Tris-HCl, pH 7.5, 5 mM MgCl2, 1 mM DTT, 2 mM ATP). Protein was extracted by the standard glass beads method and concentration of the extracts was measured and normalized using the Bradford protein assay. Equal amounts of extracts from YL1C cells expressing *AMN1*^368D^, *AMN1*^368V^ or Damn1 mutant cells were added with 2 μg N-terminal histidine tagged human ubiquitin, and the cell extracts were incubated with Myc-Ace2 (purified from YL1C cells with *AMN1* deleted) for 1 hour at 30°C. Myc-Ace2 were recovered using 20 μl of dynabeads protein G (Invitrogen, USA) that was pre-incubated with the monoclonal mouse anti-Myc (Transgene, China) antibody for 30 min at room temperature. After three washes with the lysis washing buffer (50 mM HEPES-KOH at pH 7.5, 140 mM NaCl, 1 mM EDTA, 1% Triton X-100), ubiquitylated proteins were detected using antibodies against the His epitope.

### Chromatin immuno-precipitation

Ste12 tagged with 6 tandem repeats of c-myc epitope was used in ChIP assays for both the haploid YL1C and the corresponding diploid cells. The ChIP assay was implemented according to the classic protocol [43]. In detail, cell cultures in a volume of 50 ml were grown in YPD to an optical density of OD600 = 1.0, and incubated with 1% FA for 15 minutes at room temperature to enable protein-DNA cross-linking. After addition of 125 mM glycine and incubation for a further 5 minutes at room temperature, the cells were harvested and washed three times with ice-cold 1× TBS at pH 7.5 (20 mM Tris-Cl at pH 7.5, 150 mM NaCl). The cells were pelleted and re-suspended in 1000 μl of lysis buffer (50 mM HEPES-KOH at pH 7.5, 140 mM NaCl, 1 mM EDTA, 1% Triton X-100, 0.1% Na-deoxycholate, 1 tablet of the complete inhibitor cocktail supplied by Roche) and lysed with acid-washed glass beads for 15 minutes in a vortex on full output. After removing the cell debris by centrifugation at 12000 rpm for 5 minutes at 4°C, the chromatin in the supernatant was sheared to a length of 200 bp to 500 bp using Covaris S220 (Duty Factor = 25%, Intensity Peak Incident Power = 400W, Cycles per Burst = 200, Processing Time = 20 minutes, Volume = 1ml in TC16 tubes).

Immuno-precipitation was performed with 2.5 mg (1000 μl) cell extract in 20 μl magnetic dynabeads protein G (Invitrogen, USA), which was pre-incubated with the monoclonal mouse anti-Myc (Sigma, 9E10, USA) antibody for 2 hours at room temperature. The precipitates were washed in order with lysis buffer, lysis buffer with 360 mM NaCl, washing buffer (10 mM Tris-Cl at pH 8.0, 250 mM LiCl, 0.5% NP-40, 0.5% Na-deoxycholate, 1 mM EDTA), and 1× TE at pH 7.5 using the magnetic device supplied by Toyobo (Japan). The precipitated DNA was eluted by heating in TES for 30 minutes at 65°C and de-cross linked by heating at 65°C overnight, and digesting with proteinase K (Merck, USA) for 1 hour at 37°C. DNA in the precipitate was purified using the PCR Purification Kit (QIAGEN, USA).

## Supporting information

S1 Text supplementary material and methods. (DOCX)

S1 Fig RT-qPCR profiled expression of four downstream *AMN1* regulated genes in the YL1C strain and *Δamn1 mutant*. RT-qPCR profiled expression of four downstream genes regulated by *AMN1*, *DSE1*, *DSE2*, *SCW11* and *CTS1*, in the YL1C (YL1C, blue) and the strain with *AMN1* deleted (***Δ****amn1*, green).
(TIF)

S2 Fig Cell clumping phenotype of YL1C and its derivative strains. Microscope views of cell clumping phenotype in YL1C and its derivative strains: *Δflo8*, *FLO8* deleted strain; ***Δ****flo1*, *FLO1* deleted strain.
(TIF)

S3 Fig *ACE2* mRNA levels when AMN1 is inducibly expressed. Galactose induced expression of *AMN1* under the *GAL10* promoter after 0, 2, 4 and 6 hours. RNA levels of *AMN1* (blue) and *ACE2* (green).
(TIF)

S4 Fig Amn1 inhibited post-mitotic cell separation prior to nuclear import regulation of Ace2. **(A)** Inhibited cell separation of the YL1C strain carrying continuously activated Ace2. Microscope views of the cell clumping phenotype for YL1C, YL1C carrying Ace2-AAA-2D and Ace2-AAA-F127V mutants are shown in the lower panel and RNA expression levels of *DSE1*, *DSE2*, *SCW11* and *CTS1* from RT-qPCR are shown in the upper panel. **(B)** Protein levels of endogenously expressed Ace2-AAA-2D and Ace2-AAA-F127V in YL1C and YL1C ***Δ****amn1* strains. **Both** continuously activated mutant alleles were fused to the promoter of *ACE2* gene.
(TIF)

S5 Fig *ACE2* translation efficiency in YL1C and YL1C *Δamn1* strains. RT-qPCR quantified mRNA and ribosome protected mRNA of *ACE2* and *ACT1* of the gene in YL1C or *AMN1* deleted strains. The Ct values were used to quantify the RNA fragments. **The right panel:** Primers used for the assays were listed and corresponding to the labeling of the left panel’s ordinates. rp in the figure is for ribosome profiling.
(TIF)

S6 Fig The 11-residue domain of Ace2 for Amn1 binding. **(A)** Schematic protein structure of paralogous genes *ACE2* (blue) and *SWI5* (green) (upper left), and their three homologous domains, A, B, and C. The 11-residue domain within Ace2 is highlighted by the red bar. Vertical lines indicate highly homologous segments (≥80% amino acid sequence similarity) between the two proteins. The six chimeric proteins constructed from the three homologous regions (lower left) and the corresponding cell clumping phenotypes (right). **(B)** Alignment of Ace2 orthologs among the three yeast species (*K*. *lactis*, *C*. *glabrata*, *S*. *cerevisiae*) and the Swi5 protein in *S*. *cerevisiae*. The 11-residue domain is highlighted with a yellow background. **(C)** Cell clumping phenotype observed under the microscope for strains bearing single amino acid substitutions (Ace2-R71N, Ace2-D74N, or Ace2-V78G as shown in red in (B)).
(TIF)

S7 Fig Alignment of orthologous Amn1 sequences. **(A)** The functional aspartic acid residues (D, red letters) within the conserved Leucine rich repeat domain (LxxLxLxxN/CxL, orange letters) located in the C-terminal of Amn1 of 18 yeast species. Full length of Amn1 protein sequences were downloaded from OrthoDB (http://cegg.unige.ch/), aligned globally and the phylogenetic tree was built using on the Neighbor-joining (NJ) method implemented into the software Mega4.0. Fianlly listed only the leucine rich repeat domain of 11-residue sequences for a demonstration purpose. Leucine(L)/ isoleucine(I)/alanine(A) were considered as similar residues. *Orthologous genes of *AMN1* were multi-copied in Clavispora lusitaniae. **Amn1^368D^ was used for sequence alignment. **(B)** Cell clumping phenotype of YL1C with endogenous *AMN1* replaced by *AMN1*^*Kl*^, *AMN1*^*Cg*^ *in situ* or ***Δ****amn1* mutant cells. **(C)** RNA levels of *DSE1* (blue), *DSE2* (green), *SCW11* (purple) and *CTS1* (cyan) in the YL1C strain with various genetic modifications. **(D)** Protein sequences aligned among Amn1^*S*. *cerevisiae*^ (368D), Amn1^*K*. *lactis*^ and Amn1^*C*. *glabrata*^ using Clustal W. Black (or grey) letters represent identical residues among all three (or two) species. The conserved leucine rich repeat domain shown in (A) was highlighted in orange box.
(TIF)

S1 Table Up-regulated genes involved in daughter cell separation. (DOCX)

S2 Table The 368^th^ amino acid residue of Amn1 in yeast strains with known genome sequence. (DOCX)

S3 Table A list of strains used in this study. (DOCX)

S4 Table A list of plasmids used in this study. (DOCX)
