# Supplementary material for: Amn1 governs post-mitotic cell separation in Saccharomyces cerevisiae
Source: PLoS Genet. 2018 Oct 1;14(10):e1007691. doi: 10.1371/journal.pgen.1007691 (PMC6181423; doi:10.1371/journal.pgen.1007691)
Supplement: S1 Text — (DOCX) [file pgen.1007691.s001.docx]

Supporting Information (Material and Methods) for:

***“*Amn1 Governs Post-mitotic Cell Separation in *Saccharomyces cerevisiae”***

Ou Fang ^1,3^, Xiaohua Hu^1^, Lin Wang^1^, Ning Jiang^1^, Jixuan Yang^1^, Bo Li^3^ and Zewei Luo^1,2*^

^1^ Laboratory of Population and Quantitative Genetics, Institute of Biostatistics,

School of Life Sciences, Fudan University, Shanghai 200433, China

^2^ School of Biosciences, the University of Birmingham, Birmingham B15 2TT,

United Kingdom

^3^ Department of Evolution and Ecology, School of Life Sciences, Fudan

University, Shanghai 200433, China

**Yeast Strains, Cell Clumping Phenotype and Growth Media**

For displaying yeast cell clumping phenotype, the test cells were diluted to an equivalent optical density (OD600 = 3.0) and examined in an equivalent volume of 10μl for microscope viewing. The clumping phenotype of the clumpy and non-clumpy strains was displayed under either high or low magnification in order to capture comparable numbers of cells between images. We implemented the one-step PCR-based technique to knock out or epitope tag the target genes as previously described [1], using *hphMX4* (anti-hygromycin B), *natMX4* (anti-nourseothricin), *kanMX4* (anti-G418), or *sh-ble* (anti-Zeocin) as selection markers. *PDR5* gene was knocked out in MG132 treated strains. To obtain the engineered strains carrying point mutations of Amn1 and Ace2, Ace2-Swi5 chimeras, or with Amn1 promoter lacking the binding site of a1-α2, we knocked out the corresponding regions in the genome *in situ* using the *URA3* marker and replaced with the corresponding designed fragments.

Genes of interest were overexpressed using plasmid *pGU* or *pRHON*, and yeast mating type switch was made by use of plasmid *pTetra* (see Plasmid construction and yeast cell engineering. The primer sequences for engineered yeast strains or plasmid construction are available upon request from the corresponding authors. The PEG-LiAC method was used to transform DNA fragments or vectors into yeast cells [2]. All genetic modified strains were confirmed by PCR and/or DNA sequencing experiments. Unless specified, yeast strains were grown in the standard rich medium YPD (1% yeast extract, 2% polypeptone, 2% glucose, plus 2% agar whenever necessary). In the mating type switch and gene overexpression experiments, YPR (1% yeast extract, 2% polypeptone, 2% raffinose) and YPRG (1% yeast extract, 2% polypeptone, 2% galactose, 2% raffinose) media were used. Synthetic complete medium lacking uracil (SC-U) and synthetic complete medium with added 5-fluoroorotic acids (SC+5FOA) were used for *URA3* mutagenesis experiments.

**Plasmid construction and yeast cell engineering**

All plasmids used in the present study are listed in Table S4. Plasmid *pGU* was used for high level inducible protein expression. It was generated from the plasmid *pSH47* with a little modification [3]. DNA oligos containing *Sfi* I-A (GGCCATTACGGCC) and *Sfi* I-B(GGCCGCCTCGGCC) sites were synthesized and ligated into the *BamH* I site and *Xho* I site of *pSH47*. *MYC-ACE2, MYC-ACE2**, *AMN1^368D^-FLAG* and *AMN1^368V^-FLAG* were assembled by PCR, and then inserted into *Sfi* I-A/*Sfi* I-B to generate the plasmids of interest named as *pGU-MYC-ACE2, pGU-MYC-ACE2*, pGU-AMN1^368D^-FLAG and pGU-AMN1^368V^-FLAG* respectively. These plasmids were transformed into yeast cells and the inserted genes would be inducibly expressed on exposure to galactose.

We also constructed a plasmid *pRHON* to clone and propagate target genes for constitutive overexpression. *pRHON* was derived from the plasmid *HO-Poly-KanMX4-HO* [4]. *HO-Poly-KanMX4-HO* carries long flanking sequence of the *HO* gene, which can be used for integration of a gene expression cassette into the HO locus of *S. cerevisiae*. Firstly, *natMX4* from *PAG36* [5] was used to replace the selection marker *kanMX4* at the *Bgl* II and *Sac* I sites. Yeast *ADH1* promoter with *Sfi* I-A/*Sfi* I-B sites at the 3’ terminal was amplified from the yeast genome and then inserted into the vector at the *BamH* I and *Asc* I sites. Finally, the *CYCI* terminator was amplified from *pSH47* and cloned into the *Asc* I and *Bgl* II sites. *ACE2*, *AMN1* or *STE12* ORFs were fused into the *Sfi* I-A/*Sfi* I-B sites in the corresponding expression plasmid. These plasmids propagated in *E. coli* cells were harvested and cut using *Spe* I, and then transformed into yeast cells. The positive yeast cell clones should be integrated with the gene expression cassette of interest.

*pTetra* was used for the mating type switch and for screening diploids with a/a or *α*/*α* mating type. It was modified from the plasmid *YCP50* [6]. The *P_HO_-natMX4-T_HO_* cassette was firstly amplified and assembled from the yeast genome and *PAG36* plasmid [5] through an overlap extension polymerase chain reaction (PCR), and then cloned into *YCP50* at the *EcoR* I and *BamH* I sites. The cassette *hphMX4* from *PAG32* [5] was used to replace the selection marker *URA3* via the *Sal* I and *Nsi* I restriction sites. Finally, the fragment *P_GAL10_-HO-T_cyc1_* cassette was assembled by PCR and ligated into the vector at the *Xho* I site. *pTetra* was then transformed into yeast cells. The cells were streaked out onto YPD plates with added nourseothricin after a short-time exposure on YPGAL. When cell colonies were discernible, their mating types were confirmed either through mating with standard strains of a known mating type or through PCR assay using the primers “AGTCACATCAAGATCGTTTATGG, GCACGGAATATGGGACTACTTCG and ACTCCACTTCAAGTAAGAGTTTG” [7].

**Measuring translation efficiency of *ACE2***

Translation efficiency of *ACE2* was measured using a protocol of ribosome profiling described by C. Joel McManus, but with a little modification [8]. YL1C and YL1C *Δamn1* strains were grown in 650ml cultures to an OD600 of 0.8 in YPD at 30°C. Cells were treated with cycloheximide (100μg/ml final) for 5 minutes and harvested by centrifugation. After being washed twice using polysome lysis buffer (PLB: 20mM Tris-Cl (pH8.0), 140mM KCl, 1.5ml MgCl_2_, 1% Triton X-100, 100μg/ml cycloheximide), cells were pelleted and frozen in liquid nitrogen. WCEs (whole cell extracts) were extracted using the standard glass beads method and quantified by OD260 measurement via NanoDrop. A 50 OD aliquot of WCEs were digested with 800U of RNase I (Life Technologies) for 50 minutes at room temperature with gentle rotation. The digestion was stopped by adding 200U of SUPERase-In (Life Technologies). The digested WCEs were then loaded onto a 50% sucrose cushion and centrifuged at 4°C for 4 hours at 40,000rpm using L90K with the SW60Ti rotor (Beckman). The resulting pellets were resuspended in TES (10mM Tris-Cl (pH8.0), 1mM EDTA (pH8.0), 1% SDS) and the RNA was purified by two rounds of hot phenol-chloroform extraction. The purified samples were then loaded onto a 15% Urea-Polyacrylamide gel and the gel slices corresponding to 28nt and 30nt were excised. These ribosome protected RNA oligos were then recovered and processed into sequencing libraries using the ‘NEBNext Small RNA Library Prep Set for Illumina’, following the manufacturer’s instructions. The standard mRNA-seq libraries were prepared using the TruSeq RNA Library Prep Kit. All libraries were quantified by Qubit and RT-qPCR was performed using 1ng library DNA as template. The sense primers for RT-qPCR matched to the specific genes (Figure S5) and the antisense primer “CAAGCAGAAGACGGCATACGAGAT” matched to the common sequence of the Illumina sequencing adaptor.

**Identifying the Amn1 binding domain of Ace2**

Ace2 and Swi5 amino acid sequences were aligned and three regions could be distinguished (Figure S9A): region C comprises of the most conserved zinc finger region (79.9% identity), the N-terminal region A is less conserved (23.9% identity), and the middle region B (18.3% identity) is Ace2/Swi5-specific and determines their corresponding target genes^7^. To identify the region through which *AMN1* specifically down-regulated Ace2 and inhibited cell separation, we constructed various chimeric fusions between the genic regions of Ace2 and its paralog Swi5 (Figure S9A). When the Ace2 allele of YL1C was replaced by the fused Swi5^(A)^-Ace2^(BC)^ or Swi5^(A)^-Ace2^(B)^-Swi5^(A)^ chimeras, the cells separated normally (panels 1 and 3 in Figure S4A). We therefore focused on region A and aligned the amino acid sequences of Ace2^Sc^ (NP_013232.1), Ace2^Kl^ (XP_453786) and Ace2^Cg^ (XP_449535.1). According to their sequence similarity, we detected three conserved regions, including an unannotated leucine rich domain, the NES (nuclear exit sequence) and the binding site of kinase Cbk1 (Figure S9B). We further divided region A of Ace2 into four parts, and constructed various chimeras of these sub-regions as illustrated in 4, 5 and 6 of Figure S9A. The YL1C cells carrying Ace2^(A1,A2)^-Swi5^(A3,A4)^ or Ace2^(A1,A2,A3)^-Swi5^(A4)^ do not show the clumpy phenotype (5,6 in Figure S9A), while cells carrying Ace2^(A1)^-Swi5^(A2,A3,A4)^ show the cell clumping phenotype (4 in Figure S9A). We were therefore able to narrow down the causative region by which *AMN1* inactivates Ace2 to region A2, i.e. Ace2 (69-116aa). In this region, we found a novel conserved leucine rich domain ‘ELRDLDIPLVP’ (highlighted in yellow in Figure S9B).

**Fluorescence Microscopy**

Cells carrying GFP tagged Ace2 were grown in synthetic complete medium and synchronized with α-factor arrest as described previously [12]. Cells were harvested every 20 minutes and the fluorescent images were captured with fluorescent microscopy (Olympus, IX71) with a 100X objective. Images were acquired and analyzed with software (DP2-BSW).

**Reference cited above**

1. Lorenz MC *et al. (*1995) Gene disruption with PCR products in Saccharomyces cerevisiae. *Gene* 158:113–117.
2. Gietz RD, Schiestl RH (2007) High-efficiency yeast transformation using the LiAc/SS carrier DNA/PEG method. *Nature protocols* 2:31–34.
3. Güldener U, Heck S, Fielder T, Beinhauer J, Hegemann JH (1996) A new efficient gene disruption cassette for repeated use in budding yeast. *Nucleic acids research* 24:2519–2524.
4. Voth WP, Richards JD, Shaw JM, Stillman DJ (2001) Yeast vectors for integration at the HO locus. *Nucleic acids research* 29:E59-9.
5. Goldstein AL, McCusker JH (1999) Three new dominant drug resistance cassettes for gene disruption in Saccharomyces cerevisiae. *Yeast (Chichester, England)* 15:1541–1553.
6. Rose MD, Novick P, Thomas JH, Botstein D, Fink GR (1987) A Saccharomyces cerevisiae genomic plasmid bank based on a centromere-containing shuttle vector. *Gene* 60:237–243.
7. Huxley C, Green ED, Dunham I (1990) Rapid assessment of S. cerevisiae mating type by PCR. *Trends in genetics : TIG* 6:236.
8. McManus CJ, May GE, Spealman P, Shteyman A (2014) Ribosome profiling reveals post-transcriptional buffering of divergent gene expression in yeast. *Genome research* 24:422–430.
