# Supplementary figures and images for: Amn1 governs post-mitotic cell separation in Saccharomyces cerevisiae
Source: PLoS Genet. 2018 Oct 1;14(10):e1007691. doi: 10.1371/journal.pgen.1007691 (PMC6181423; doi:10.1371/journal.pgen.1007691)

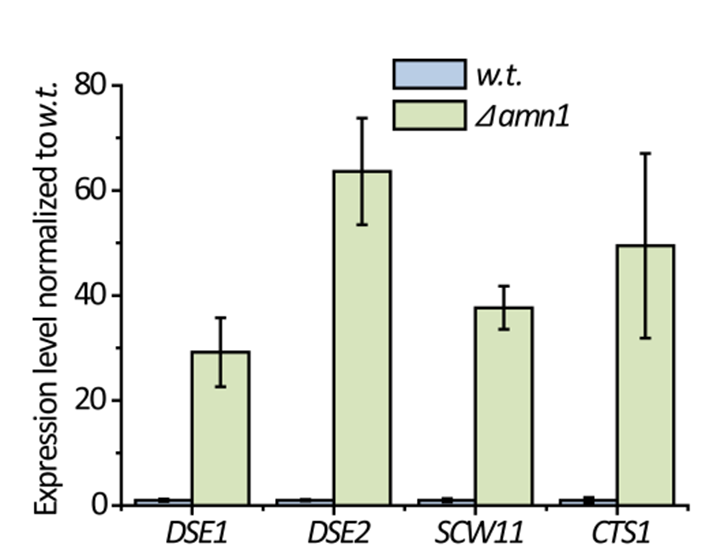

Supplement: S1 Fig — RT-qPCR profiled expression of four downstream genes regulated by AMN1, DSE1, DSE2, SCW11 and CTS1, in the YL1C (YL1C, blue) and the strain with AMN1 deleted (Δamn1, green). (TIF) [file pgen.1007691.s002.tif]

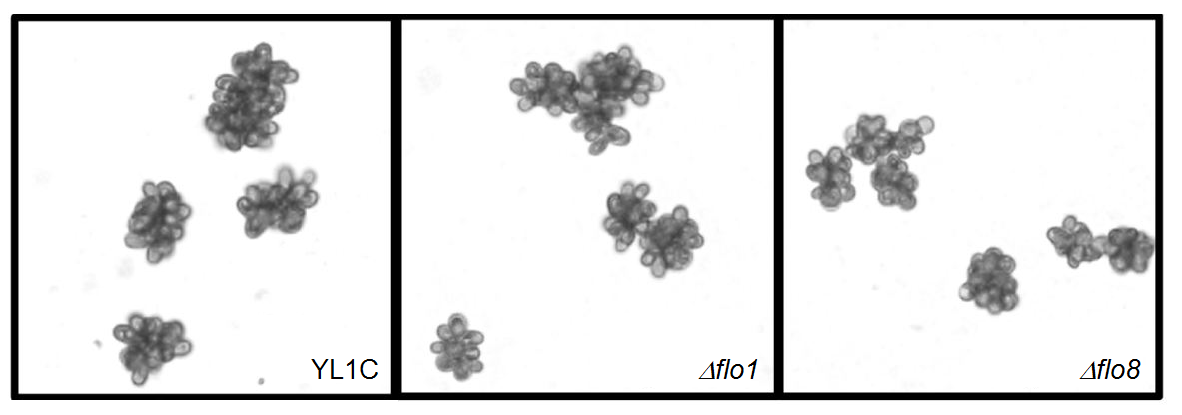

Supplement: S2 Fig — Microscope views of cell clumping phenotype in YL1C and its derivative strains: Δflo8, FLO8 deleted strain; Δflo1, FLO1 deleted strain. (TIF) [file pgen.1007691.s003.tif]

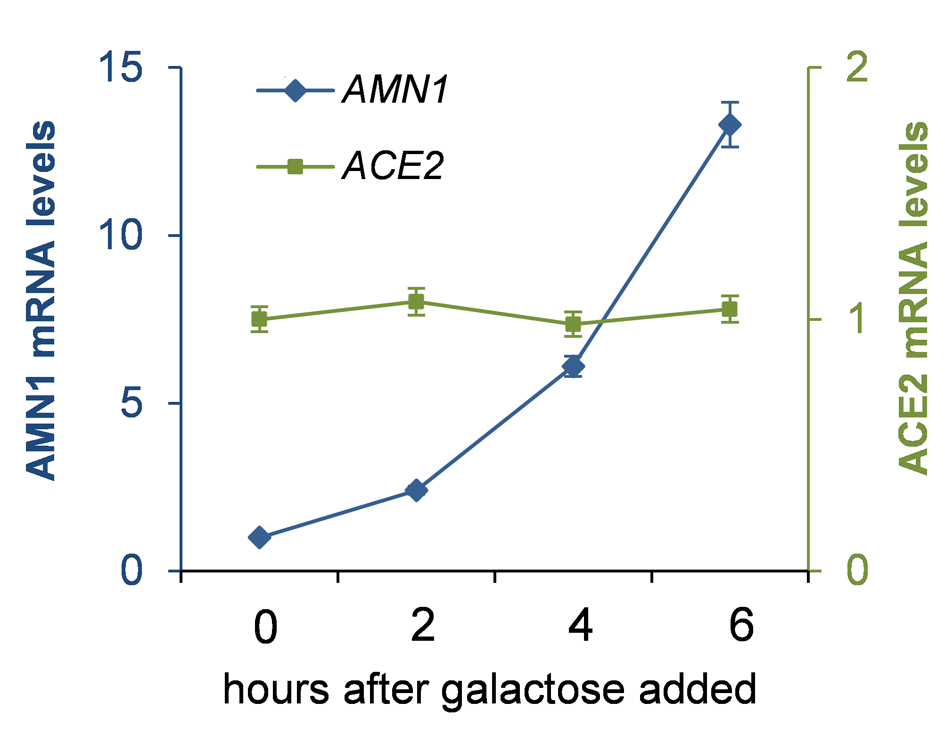

Supplement: S3 Fig — Galactose induced expression of AMN1 under the GAL10 promoter after 0, 2, 4 and 6 hours. RNA levels of AMN1 (blue) and ACE2 (green). (TIF) [file pgen.1007691.s004.tif]

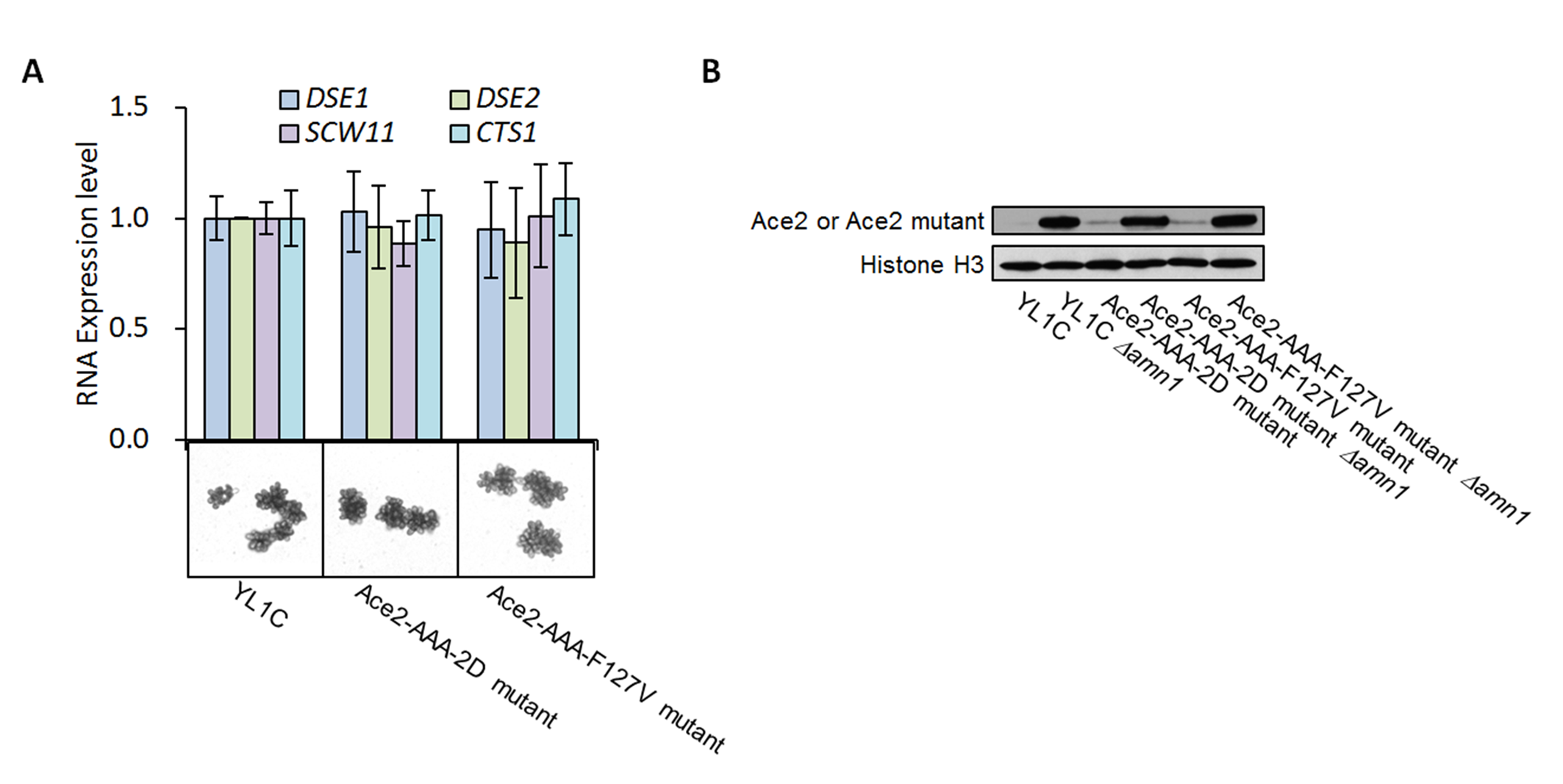

Supplement: S4 Fig — (A) Inhibited cell separation of the YL1C strain carrying continuously activated Ace2. Microscope views of the cell clumping phenotype for YL1C, YL1C carrying Ace2-AAA-2D and Ace2-AAA-F127V mutants are shown in the lower panel and RNA expression levels of DSE1, DSE2, SCW11 and CTS1 from RT-qPCR are shown in the upper panel. (B) Protein levels of endogenously expressed Ace2-AAA-2D and Ace2-AAA-F127V in YL1C and YL1C Δamn1 strains. Both continuously activated mutant alleles were fused to the promoter of ACE2 gene. (TIF) [file pgen.1007691.s005.tif]

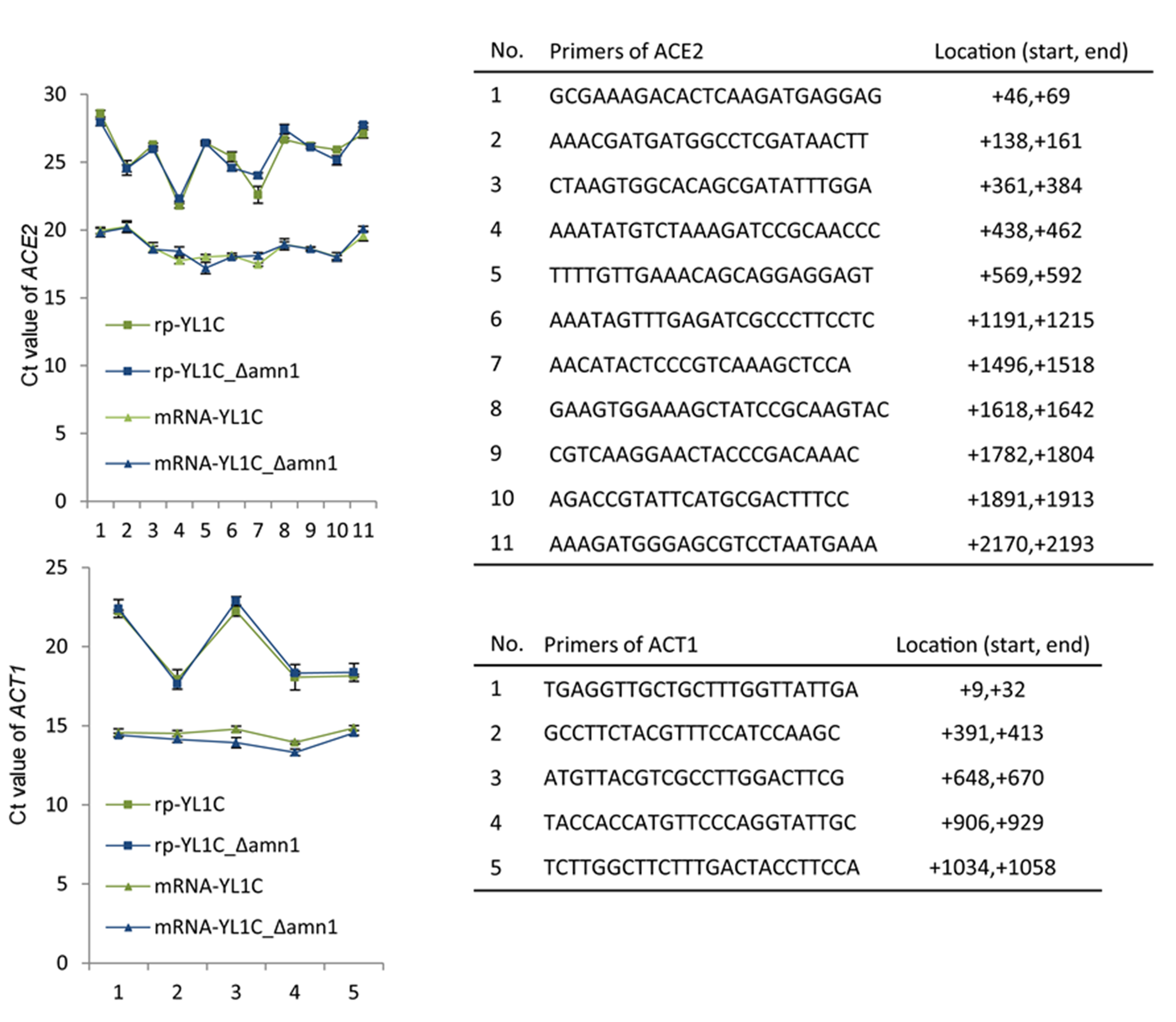

Supplement: S5 Fig — RT-qPCR quantified mRNA and ribosome protected mRNA of ACE2 and ACT1 of the gene in YL1C or AMN1 deleted strains. The Ct values were used to quantify the RNA fragments. The right panel: Primers used for the assays were listed and corresponding to the labeling of the left panel’s ordinates. rp in the figure is for ribosome profiling. (TIF) [file pgen.1007691.s006.tif]

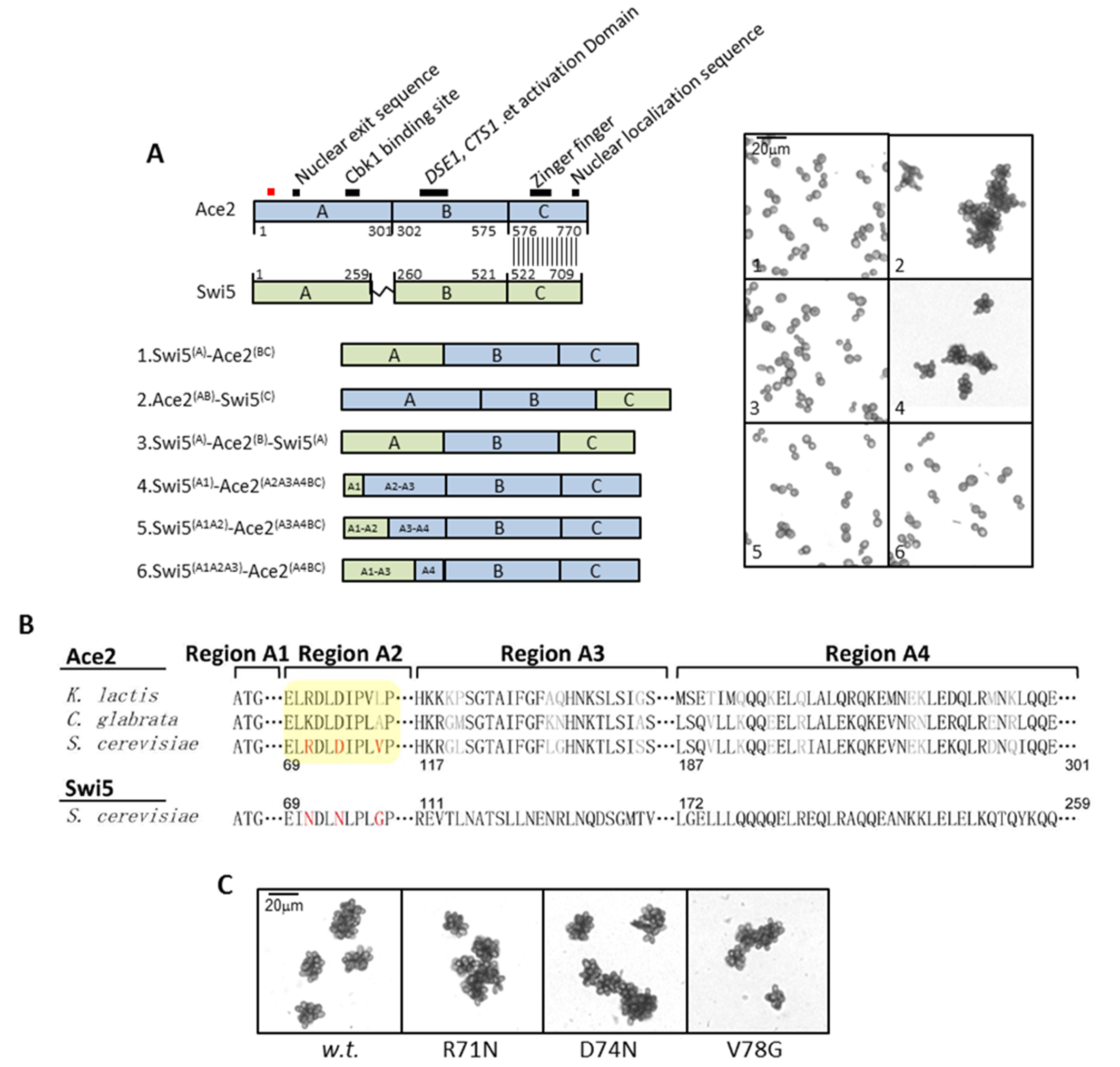

Supplement: S6 Fig — (A) Schematic protein structure of paralogous genes ACE2 (blue) and SWI5 (green) (upper left), and their three homologous domains, A, B, and C. The 11-residue domain within Ace2 is highlighted by the red bar. Vertical lines indicate highly homologous segments (≥80% amino acid sequence similarity) between the two proteins. The six chimeric proteins constructed from the three homologous regions (lower left) and the corresponding cell clumping phenotypes (right). (B) Alignment of Ace2 orthologs among the three yeast species (K. lactis, C. glabrata, S. cerevisiae) and the Swi5 protein in S. cerevisiae. The 11-residue domain is highlighted with a yellow background. (C) Cell clumping phenotype observed under the microscope for strains bearing single amino acid substitutions (Ace2-R71N, Ace2-D74N, or Ace2-V78G as shown in red in (B)). (TIF) [file pgen.1007691.s007.tif]

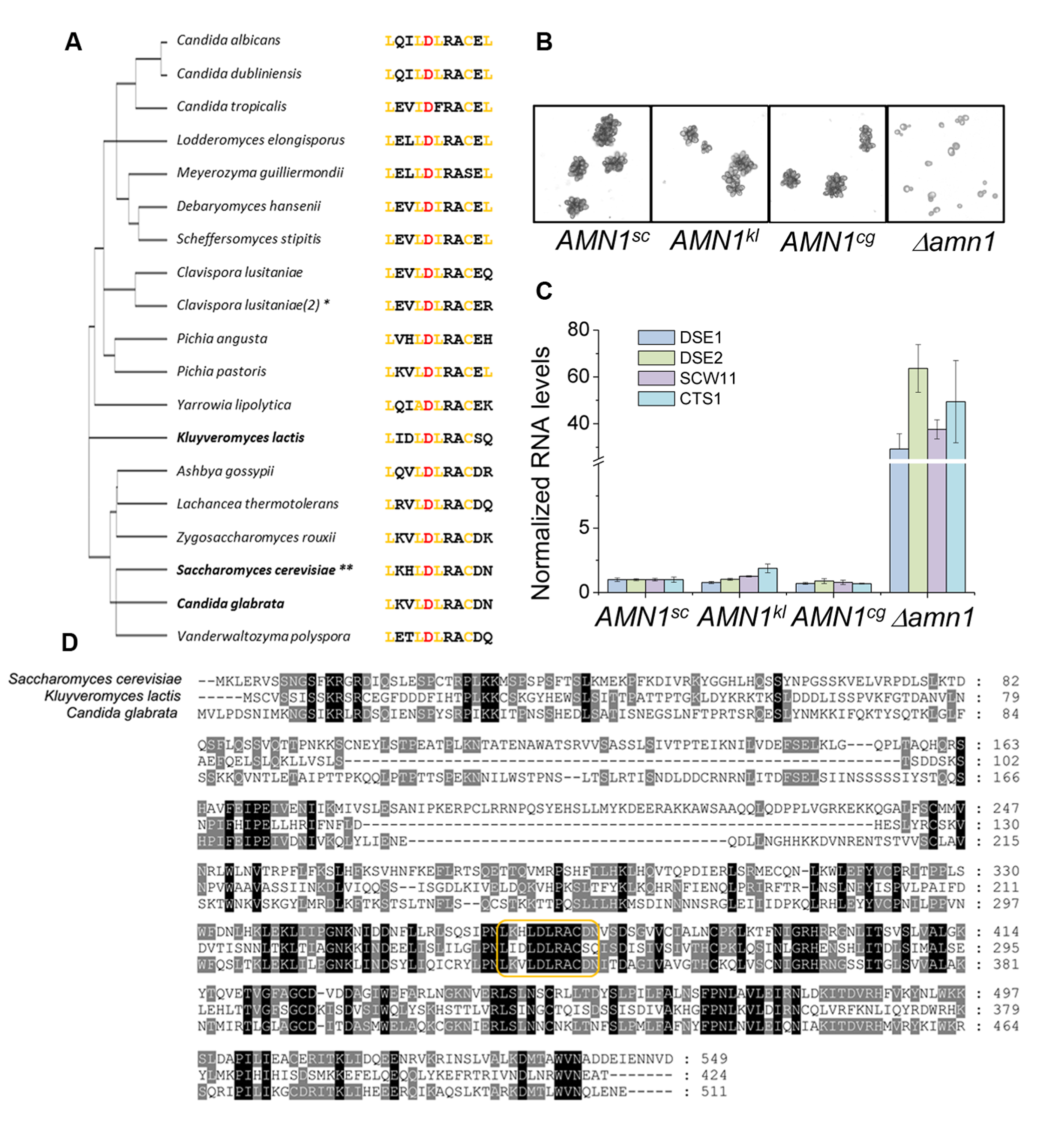

Supplement: S7 Fig — (A) The functional aspartic acid residues (D, red letters) within the conserved Leucine rich repeat domain (LxxLxLxxN/CxL, orange letters) located in the C-terminal of Amn1 of 18 yeast species. Full length of Amn1 protein sequences were downloaded from OrthoDB (http://cegg.unige.ch/), aligned globally and the phylogenetic tree was built using on the Neighbor-joining (NJ) method implemented into the software Mega4.0. Fianlly listed only the leucine rich repeat domain of 11-residue sequences for a demonstration purpose. Leucine(L)/ isoleucine(I)/alanine(A) were considered as similar residues. *Orthologous genes of AMN1 were multi-copied in Clavispora lusitaniae. **Amn1368D was used for sequence alignment. (B) Cell clumping phenotype of YL1C with endogenous AMN1 replaced by AMN1Kl, AMN1Cg in situ or Δamn1 mutant cells. (C) RNA levels of DSE1 (blue), DSE2 (green), SCW11 (purple) and CTS1 (cyan) in the YL1C strain with various genetic modifications. (D) Protein sequences aligned among Amn1S. cerevisiae (368D), Amn1K. lactis and Amn1C. glabrata using Clustal W. Black (or grey) letters represent identical residues among all three (or two) species. The conserved leucine rich repeat domain shown in (A) was highlighted in orange box. (TIF) [file pgen.1007691.s008.tif]
