# Supplementary material for: Amn1 governs post-mitotic cell separation in Saccharomyces cerevisiae
Source: PLoS Genet. 2018 Oct 1;14(10):e1007691. doi: 10.1371/journal.pgen.1007691 (PMC6181423; doi:10.1371/journal.pgen.1007691)
Supplement: S1 Table — (DOCX) [file pgen.1007691.s009.docx]

| **Table S1 Up-regulated genes involved in daughter cell separation** | | | | |
| --- | --- | --- | --- | --- |
| **Gene** | **F. change^*^** | **P value** | **Function^**^** |  |
| ***CTS1*** | 23.8 | 0 | Endochitinase; required for cell separation after mitosis; transcriptional activation during the G1 phase of the cell cycle is mediated by transcription factor Ace2p |  |
| ***DSE1*** | 85.1 | 0 | Daughter cell-specific protein; may regulate cross-talk between the mating and filamentation pathways; deletion affects cell separation after division and sensitivity to alpha-factor and drugs affecting the cell wall. |  |
| ***DSE2*** | 7.5 | 9.70E-11 | Daughter cell-specific secreted protein with similarity to glucanases; degrades cell wall from the daughter side causing daughter to separate from mother; expression is repressed by cAMP |  |
| ***DSE3*** | 3.8 | 1.14E-05 | Daughter cell-specific protein, may help establish daughter cell fate; relocalizes from bud neck to cytoplasm upon DNA replication stress |  |
| ***DSE4*** | 4.0 | 5.82E-06 | Daughter cell-specific secreted protein with similarity to glucanases; degrades cell wall from the daughter side causing daughter to separate from mother |  |
| ***EGT2*** | 3.4 | 8.46E-06 | Glycosylphosphatidylinositol (GPI)-anchored cell wall endoglucanase; required for proper cell separation after cytokinesis; expression is activated by Swi5p and tightly regulated in a cell cycle-dependent manner |  |
| ***SCW11*** | 19.1 | 0 | Cell wall protein with similarity to glucanases; may play a role in conjugation during mating based on its regulation by Ste12p |  |
| * Fold change in the *AMN1* deleted strain when compared to YL1C.  **Gene function annotated from the *Saccharomyces* Genome Database (http://www.yeastgenome.org) | | | | |
