# Supplementary material for: Amn1 governs post-mitotic cell separation in Saccharomyces cerevisiae
Source: PLoS Genet. 2018 Oct 1;14(10):e1007691. doi: 10.1371/journal.pgen.1007691 (PMC6181423; doi:10.1371/journal.pgen.1007691)
Supplement: S2 Table — (DOCX) [file pgen.1007691.s010.docx]

| **Table S2 The 368^th^ amino acid residue of Amn1 in yeast strains with known genome sequence*** | |
| --- | --- |
| ***AMN1^368D^***  **(allele of YL1C)** | ***AMN1^368V^***  **(allele of s288c)** |
| \| 10560-6B \| \| --- \| \| AWRI1631 \| \| AWRI796 \| \| BC187 \| \| CBS 7960 \| \| CLIB215 \| \| CLIB324 \| \| D273-10B \| \| DBVPG6044 \| \| EC1118 \| \| EC9-8 \| \| FostersB \| \| JAY291 \| \| K11 \| \| Kyokai7 \| \| L1528 \| \| M22 \| \| PW5 \| \| RedStar \| \| RM11-1A \| \| Sigma1278b \| \| SK1 \| \| T7 \| \| UC5 \| \| UWOPS05_217_3 \| \| VL3 \| \| Y55 \| \| YJM269 \| \| YJM339 \| \| YJM789 \| \| YPS128 \| \| YPS163 \| \| YS9 \| \| ZTW1 \| | \| CEN.PK113-7D \| \| --- \| \| CEN.PK2-1Ca \| \| FL100 \| \| FY1679** \| \| JK9-3d** \| \| S288C \| \| SEY6210 \| \| W303** \| \| X2180-1A** \| \| YPH499** \| \| BY4741** \| \| BY4742** \| |
| *Data is from SGD (http://www.yeastgenome.org/)  ** These strains are Isogenic to s288c, or s288c derivative. | |
