# Supplementary material for: Amn1 governs post-mitotic cell separation in Saccharomyces cerevisiae
Source: PLoS Genet. 2018 Oct 1;14(10):e1007691. doi: 10.1371/journal.pgen.1007691 (PMC6181423; doi:10.1371/journal.pgen.1007691)
Supplement: S3 Table — (DOCX) [file pgen.1007691.s011.docx]

| **Table S3 A list of strains used in this study** | |  |
| --- | --- | --- |
| **Strains** | **Relevant genotypes** | **Source** |
| ***YL1C*** | ***MATα ho::kanMX4 ura3Δ0*** | Hu et al.(2007) |
|  | ***From YFO001 to YFO037 strains were isogenic to YL1C*** |  |
| *YFO001* | *MATα amn1Δ::hphMX4* | This study |
| *YFO002* | *MATα flo1Δ::hphMX4* | This study |
| *YFO003* | *MATα flo8Δ::hphMX4* | This study |
| *YFO004* | *MATα ACE2::6XMYC-ACE2* | This study |
| *YFO005* | *MATα AMN1::AMN1-3XFLAG-natMX4* | This study |
| *YFO006* | *MATα ACE2::6XMYC-ACE2 AMN1:: AMN1-3XFLAG -natMX4* | This study |
| *YFO007* | *MATα ACE2::6XMYC-ACE2 amn1Δ::hphMX4* | This study |
| *YFO008* | *MATα ACE2::6XMYC-ACE2 pGU-AMN1-368D-3XFLAG* | This study |
| *YFO009* | *MATα ACE2::6XMYC-ACE2 pGU-AMN1-368V-3XFLAG* | This study |
| *YFO010* | *MATα ace2Δ::sh-ble AMN1::AMN1-3XFLAG-hphMX4 pGU-6XMYC-ACE2* | This study |
| *YFO011* | *MATα ace2Δ::sh-ble amn1Δ::hphMX4 pGU-6XMYC-ACE2* | This study |
| *YFO012* | *MATα ace2Δ::sh-ble AMN1::AMN1-3XFLAG-hphMX4 pdr5Δ::natMX4 pGU-6XMYC-Ace2* | This study |
| *YFO013* | *MATα ace2::sh-ble amn1Δ::hphMX4 pdr5Δ::natMX4 pGU-6XMYC-ACE2* | This study |
| *YFO014* | *MATα ace2::sh-ble AMN1::AMN1-368V-3XFLAG-hphMX4 pdr5Δ::natMX4 pGU-6XMYC-ACE2* | This study |
| *YFO015* | *MATα ACE2::6XMYC-ACE2 AMN1::AMN1-3XFLAG-hphMX4 ubi4Δ::natMX4* | This study |
| *YFO016* | *MATα ACE2::6XMYC-ACE2 AMN1::AMN1-3XFLAG -hphMX4 pdr5Δ::natMX4* | This study |
| *YFO017* | *MATα ACE2::6XMYC-ACE2 amn1Δ:: hphMX4 ho::P_ADH1_-AMN1-368D-3XFLAG-T_CYC1_-natMX4* | This study |
| *YFO018* | *MATα ACE2::6XMYC-ACE2 amn1Δ:: hphMX4 ho::P_ADH1_-AMN1-Δ(496-789)-3XFLAG-T_CYC1_-natMX4* | This study |
| *YFO019* | *MATα ACE2::6XMYC-ACE2 amn1Δ:: hphMX4 ho::P_ADH1_-AMN1-Δ(496-552)&(721-789)-3XFLAG-T_CYC1_-natMX4* | This study |
| *YFO020* | *MATα CDC53::CDC53-6XMYC-natMX4 AMN1::AMN1-3XFLAG -hphMX4* | This study |
| *YFO021* | *MATα SKP1::SKP1-6XMYC-natMX4 AMN1::AMN1-3XFLAG -hphMX4* | This study |
| *YFO022* | *MATα CDC53::CDC53-8XHA-natMX4 SKP1::SKP1-6XMYC-natMX4 AMN1::AMN1-3XFLAG-URA3* | This study |
| *YFO023* | *MATα SWI5::6XMYC-SWI5* | This study |
| *YFO024* | *MATα SWI5::6XMYC-SWI5 amn1Δ::hphMX4* | This study |
| *YFO025* | *MATα ace2Δ::sh-ble AMN1::AMN1-3XFLAG-hphMX4 pdr5Δ::natMX4 pGU-6XMYC-ACE2 (L70I & R71N & D74N & I75L & V78G)* | This study |
| *YFO026* | *MATα ACE2::6XMYC-ACE2(L70I & R71N & D74N & I75L & V78G)* | This study |
| *YFO027* | *MATα TEM1::TEM1-6XMYC-natMX4 AMN1::AMN1-3XFLAG-hphMX4* | This study |
| *YFO028* | *MATα TEM1::TEM1-6XMYC-natMX4 CDC15::CDC15-3XFLAG-hphMX4 amn1Δ::URA3* | This study |
| *YFO029* | *MATα TEM1::TEM1-6XMYC-natMX4 CDC15::CDC15-3XFLAG-hphMX4 ho::P_ADH1_-AMN1-3XFLAG-T_CYC1_-URA3 amn1Δ::kanMX4* | This study |
| *YFO030* | *MATα TEM1::TEM1-6XMYC-natMX4 CDC15::CDC15-FlAG-hphMX4 ho::P_ADH1_-AMN1(368V)-3XFLAG-T_CYC1_-URA3 amn1Δ::kanMX4* | This study |
| *YFO031* | *MATα AMN1::AMN1-K.lactis* | This study |
| *YFO032* | *MATα AMN1::AMN1-C.glabrata* | This study |
| *YFO033* | *MATα ace2Δ::sh-ble* | This study |
| *YFO034* | *MATα ace2Δ::sh-ble ste12Δ::natMX4* | This study |
| *YFO035* | *MATα ste12Δ::natMX4* | This study |
| *YFO036* | *MATα amn1-UAS(-1~-1000bp)Δ::natMX4* | This study |
| *YFO037* | *MATα STE12::STE12-6XMYC-natMX4* | This study |
|  | ***From YFO038 to YFO043 strains were isogenic to YL1C but MATa*** |  |
| *YFO038* | *MATa bar1Δ::natMX4 ACE2::ACE2-GFP-hphMX4* | This study |
| *YFO039* | *MATa bar1Δ::natMX4 amn1Δ::sh-ble ACE2::ACE2-GFP-hphMX4* | This study |
| *YFO040* | *MATa bar1Δ::natMX4 ACE2::ACE2(F127V & T575A & S701A & S714A)-GFP-hphMX4* | This study |
| *YFO041* | *MATa bar1Δ::natMX4 ACE2::ACE2(S122D & S137D & T575A & S701A & S714A)-GFP-hphMX4* | This study |
| *YFO042* | *MATa bar1Δ::natMX4 ACE2::MYC-ACE2 AMN1::AMN1-3XFLAG-hphMX4* | This study |
| *YFO043* | *MATa bar1Δ::natMX4 ACE2::MYC-ACE2 amn1Δ::hphMX4* | This study |
|  | ***From YFO044 to YFO055 were isogenic to YL1C but diploidized by YCP50-HO*** |  |
| *YFO044* | *Diploid YL1C MATa/α* | This study |
| *YFO045* | *MATa/α ACE2::MYC-ACE2/ACE2::MYC-ACE2 AMN1::AMN1-3XFLAG-hphMX4/ AMN1::AMN1-3XFLAG-hphMX4* | This study |
| *YFO046* | *MATa/α ACE2::MYC-ACE2/ACE2::MYC-ACE2 amn1::hphMX4/amn1::hphMX4 ho::P_ADH1_-AMN1-368D-3XFLAG-T_CYC1_-natMX4/ ho::P_ADH1_-AMN1-368D-3XFLAG-T_CYC1_-natMX4* | This study |
| *YFO047* | *Diploid YL1C MATa/a* | This study |
| *YFO048* | *Diploid YL1C MATα/α* | This study |
| *YFO049* | *MATa/α AMN1 UAS(-474~-483bp)Δ::0* | This study |
| *YFO050* | *MATa/α AMN1 UAS(-812~-823bp)Δ::0* | This study |
| *YFO051* | *MATa/α AMN1 UAS(-474~-483bp & -812~823bp)Δ::0* | This study |
| *YFO052* | *MATa/α ace2Δ::sh-ble/ace2Δ::sh-ble* | This study |
| *YFO053* | *MATa/α ace2Δ::sh-ble/ace2Δ::sh-ble ste12Δ::natMX4/ace2Δ::hphMX4* | This study |
| *YFO054* | *MATa/α STE12::STE12-6XMYC-natMX4/STE12::STE12-6XMYC-natMX4* | This study |
| *YFO055* | *MATa/α AMN1 UAS(-1~-1000bp)Δ::natMX4/AMN1 UAS(-1~-1000bp)Δ::natMX4* | This study |
| ***W303-1b*** | ***MATα leu2-3,112 trp1-1 can1-100 ura3-1 ade2-1 his3-11,15*** | ATCC:200060 |
|  | ***YFO056 & YFO057 were isogenic from W303-1b*** |  |
| *YFO056* | *MATα ho::P_ADH1-_AMN1-368D-3XFLAG-T_CYC1_-natMX4* | This study |
| *YFO057* | *MATα ho::P_ADH1_-AMN1-368V-3XFLAG-T_CYC1_-natMX4* | This study |
| ***S288C*** | ***MATα SUC2 gal2 mal2 mel flo1 flo8-1 hap1 ho bio1 bio6*** | ATCC:204508 |
|  | ***YFO058 & YFO059 were isogenic from S288C*** |  |
| *YFO058* | *MATα ho::P_ADH1_-AMN1-368D-3XFLAG-T_CYC1_-natMX4* | This study |
| *YFO059* | *MATα ho::P_ADH1_-AMN1-368V-3XFLAG-T_CYC1_-natMX4* | This study |
