# Supplementary material for: Amn1 governs post-mitotic cell separation in Saccharomyces cerevisiae
Source: PLoS Genet. 2018 Oct 1;14(10):e1007691. doi: 10.1371/journal.pgen.1007691 (PMC6181423; doi:10.1371/journal.pgen.1007691)
Supplement: S4 Table — (DOCX) [file pgen.1007691.s012.docx]

| **Table S4 A list of plasmids used in this study** | |  |
| --- | --- | --- |
| **plasmids** | **Characteristic** | **Source** |
| *pGU-AMN1-368D-FLAG* | Inducibly expressed Amn1^368D^-Flag | This study |
| *pGU-AMN1-368V-FLAG* | Inducibly expressed Amn1^368V^-Flag | This study |
| *pGU-MYC-ACE2* | Inducibly expressed Myc-Ace2 | This study |
| *pGU-MYC-ACE2** | Inducibly expressed Myc-Ace2* | This study |
| *pRHON-AMN1(368D)-FLAG* | *Overexpressed AMN1(368D)-FLAG at HO locus* | This study |
| *pRHON-AMN1(368V)-FLAG* | *Overexpressed AMN1(368V)-FLAG at HO locus* | This study |
| *pRHON-AMN1(ΔFbox)-1* | *Overexpressed AMN1-Δ(496-789) -FLAG at ho locus* | This study |
| *pRHON-AMN1(ΔFbox)-2* | *Overexpressed AMN1-Δ(496-552)&(721-789)-FLAG at ho locus* | This study |
| *pRHON-AMN1-Kl* | *Overexpressed AMN1^Kl^-FLAG at HO locus* | This study |
| *pRHON-AMN1-Cg* | *Overexpressed AMN1^Cg^ -FLAG at HO locus* | This study |
| *pRHON-STE12-MYC* | *Overexpressed STE12-MYC at HO locus* | This study |
| *pTetra* | Highly efficiently transform a/alpha diploids to a/a or alpha/alpha diploids. | This study |
